# Chromatin: the old and young of it

**DOI:** 10.3389/fmolb.2023.1270285

**Published:** 2023-10-09

**Authors:** Felicity J. Emerson, Siu Sylvia Lee

**Affiliations:** Lee Lab, Department of Molecular Biology and Genetics, Cornell University, Ithaca, NY, United States

**Keywords:** chromatin, aging, longevity, histone modifications, *C. elegans*

## Abstract

Aging affects nearly all aspects of our cells, from our DNA to our proteins to how our cells handle stress and communicate with each other. Age-related chromatin changes are of particular interest because chromatin can dynamically respond to the cellular and organismal environment, and many modifications at chromatin are reversible. Changes at chromatin occur during aging, and evidence from model organisms suggests that chromatin factors could play a role in modulating the aging process itself, as altering proteins that work at chromatin often affect the lifespan of yeast, worms, flies, and mice. The field of chromatin and aging is rapidly expanding, and high-resolution genomics tools make it possible to survey the chromatin environment or track chromatin factors implicated in longevity with precision that was not previously possible. In this review, we discuss the state of chromatin and aging research. We include examples from yeast, *Drosophila*, mice, and humans, but we particularly focus on the commonly used aging model, the worm *Caenorhabditis elegans*, in which there are many examples of chromatin factors that modulate longevity. We include evidence of both age-related changes to chromatin and evidence of specific chromatin factors linked to longevity in core histones, nuclear architecture, chromatin remodeling, and histone modifications.

## 1 Introduction

While aging affects nearly all beings, the rate of aging varies widely between species and even between individuals of the same species. In humans, studies from identical twins show us that only 20%–30% of aging can be attributed to genetics alone (Pal and Tyler, 2016). Even in laboratory settings with *C. elegans* worms that are nearly genetically identical, an individual wildtype (WT) worm can live twice as long as one of its siblings. Organisms with castes such as bees and ants show us genetically similar individuals within a species can have wildly different behavioral patterns and lifespan trajectories, as queen bees can live 100 times longer than worker bees (Remolina and Hughes, 2008). Cells taken from humans as old as 101 years of age have been successfully reprogrammed into a youthful stem cell-like state in the lab, indicating that genetics is not a limiting factor for rejuvenation (Lapasset et al., 2011; Mertens et al., 2015). To understand these phenomena, we must look beyond the genetic code to understand the broader context of how cells, tissues, and organisms interpret their genomes depending on their cellular and organismal environments.

Chromatin provides an interface between genetic information and the environment, allowing an individual’s experiences to shape the course of their life from within their cells. In order to store and protect genetic material, DNA is wrapped around histone proteins inside the nucleus, and this bundle of DNA and histones is termed chromatin. Histones do more than simply package DNA however, as the level of accessibility versus condensation of chromatin can impact the availability of DNA to binding by transcriptional machinery and therefore the expression of genes (Chen et al., 2021). The tails of histones are also frequently adorned with post-translational modifications in the guise of small chemical compounds such as methyl or acetyl groups. Histone marks are placed by proteins termed “writers”, decoded by “readers”, and removed by “erasers” (Biswas and Rao, 2018). Many histone modifications are placed co-transcriptionally (Eissenberg and Shilatifard, 2006; Saunders et al., 2006; Fong et al., 2017), and the position and type of modification often correlates with the transcriptional status of the nearby genes (Kouzarides, 2007). Histone modifications and the proteins that regulate them are often required for gene expression, however recent evidence suggests that histone modifications themselves do not directly regulate transcription, but could contribute to altering the surrounding chromatin environment and recruiting various readers and chromatin regulators (Morgan and Shilatifard, 2020; Wang Z. et al., 2022). Besides post-translational modifications, histones can also be modified by chromatin remodelers, proteins that can slide histones along DNA or remove them (Clapier and Cairns, 2009), thereby changing the chromatin structure. DNA itself can also be modified by methyl groups, and the methylation state of DNA correlates with gene expression (Unnikrishnan et al., 2019).

**FIGURE 1:**
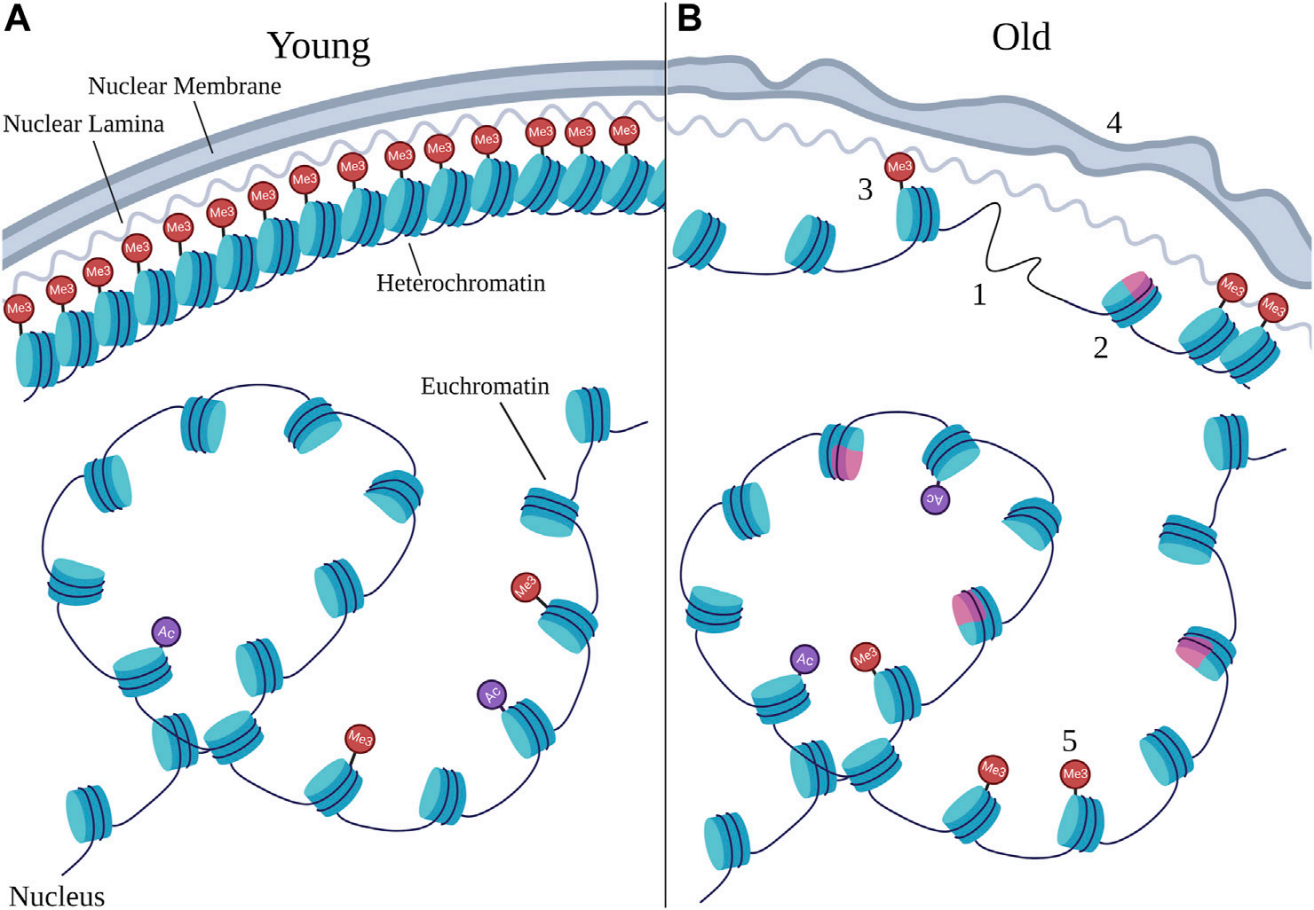
Age-related chromatin changes. Schematic representing the types of changes that can occur at chromatin during aging by comparing young nuclei **(A)** to old nuclei **(B)**. Chromatin is typically organized with dense heterochromatin anchored to the nuclear periphery and euchromatin in the nuclear interior, however the structure at heterochromatin as well as the organization of euchromatin can change during aging. Numbers denote specific age-related changes evident in old nuclei that are discussed in this review, many of which can result from changes in chromatin remodeling. 1) loss of core histones; 2) changes in histone variants, with the pink color representing a non-canonical variant; 3) loss of heterochromatin; 4) deterioration in nuclear morphology; 5) changes in site-specific histone marks. Particular changes in histone marks are depicted in Figure 2. “Ac” represents histone acetylation marks and “Me3” represents histone methylation marks, with those at the nuclear periphery representing H3K9me3 and H3K27me3, and those in the nuclear interior representing H3K4me3. Note that the changes depicted here represent the variety of changes detailed in this review, and do not necessarily always co-occur in every cell during aging.

Chromatin and aging are intricately linked. While human twins share the same genetics, their chromatin modifications diverge during aging (Cheung et al., 2018). Likewise, long-lived queen bees possess differences in DNA methylation compared to short-lived workers (Wang et al., 2020). Epigenetic changes are one of the hallmarks of aging (López-Otín et al., 2013), and changes at chromatin including alteration of core histones, loss of heterochromatin, nuclear morphology defects, and altered patterns of histone modifications, have been identified in aged samples compared to young ones (See Figure 1). Further evidence comes mainly from model organisms, in which experimental manipulation demonstrates that alteration of specific chromatin factors, such as histone modifiers or chromatin remodelers, affects longevity. In this review, we will discuss progress in the field of chromatin and aging, including both alterations at chromatin that occur during aging, and evidence that specific chromatin factors modulate longevity. The experimental evidence will focus on the well-characterized aging model *C. elegans*, which has highly conserved chromatin factors and modifications (Cui and Han, 2007), and has been used extensively in aging research with many well-defined aging pathways. We will also include examples from yeast, flies, mice, and humans in our discussion.

## 2 Changes to core histones

### 2.1 Core histone protein levels

The chromatin fiber consists of DNA wrapped around a histone octamer, an assembled subunit of eight core histone proteins: two each of histones H2A, H2B, H3, and H4 (Kouzarides, 2007). Studies in various organisms have identified differential expression of these core histone proteins during aging, with decreased expression typically seen in aged samples. The loss of core histone proteins could result in inappropriately accessible DNA due to inadequate packaging, which could lead to aberrant gene expression and increased susceptibility to DNA damage (Pal and Tyler, 2016). The most well-characterized example of histone loss during aging is in the budding yeast, *Saccharomyces cerevisiae*, where histone protein levels are decreased by approximately half during replicative aging (Dang et al., 2009; Feser et al., 2010), possibly due to the activation of the DNA damage checkpoint and genome missegregation in aged cells (Crane et al., 2019). This is despite the global increase in transcription that is observed in aging yeast cells, where nearly all RNA transcripts, including transcripts that code for histone proteins, are increased during replicative aging (Hu et al., 2014). Since increasing core histone protein levels during aging extends lifespan in yeast (Feser et al., 2010), the loss of histone proteins would seem to be a cause of replicative aging.

Similarly, H3 protein levels have been shown to decrease with aging in somatic cells of *C. elegans* (Ni et al., 2012) and in bulk analysis from male *Drosophila* (Larson et al., 2012), supporting a global decrease of core histone proteins with aging in multiple organisms. However, the genome-wide distribution of H3 protein levels in *C. elegans* somatic cells are well-correlated between young and old animals (Pu et al., 2018; Li et al., 2021), revealing no major changes in H3 occupancy during somatic aging in the worm. This suggests that H3 loss during aging may reflect a change in the free histone pool rather than occupancy of H3 at chromatin itself.

Overexpression of the histone H4 can increase lifespan in *C. elegans*, and H4 is required for a model of longevity induced by overexpression of heat shock factor 1 (HSF-1) (Sural et al., 2020). Similarly, mild histone stress via knockdown of H2A histone genes (with *his-3* RNAi) led to increased levels of H2B and H3 in a likely compensatory mechanism and a mildly extended lifespan in *C. elegans*. The extension required the chromatin remodeler ISW-1 (See “Chromatin Remodeling” section below) and HSF-1, and operated in the same genetic pathway as mitochondrial-mediated longevity (via *cco-1* RNAi) (Matilainen et al., 2017).

Although many studies have reported reduced levels of core histone proteins in mammalian aging and senescence (O’Sullivan et al., 2010; Liu B. et al., 2013; Ivanov et al., 2013; Shah et al., 2013), a recent comprehensive analysis that tested H2B and H3 protein levels in aged male mouse livers and cerebellum found no evidence of age-related decreases in core histone levels (Chen et al., 2020). Indeed, an analysis of chromatin immunoprecipitation sequencing (ChIP-seq) profiling (Johnson et al., 2007) of H3 occupancy with age in the male mouse heart, liver, cerebellum, olfactory bulb, and primary neural stem cell cultures derived from the subventricular zone found that fewer than 2% of detected nucleosomes exhibited a change in occupancy in any cell type during aging (Chen et al., 2020). Coupled with the absence of large-scale changes in histone proteins detected by Western blot, the data support largely stable H3 levels and distribution during mouse aging, similar to the findings in *C. elegans* genome-wide profiling experiments.

These varied results emphasize the importance of careful cell-type specific analysis, as dramatic changes observed in one organism or one cell type may not hold true for a whole animal or diverse organisms. Additionally, incorporating both bulk level analysis like immunoblotting along with whole-genome occupancy-level data like ChIP-seq will be important to form a complete picture of how core histones change with age in diverse systems. However, as there are likely minor changes in histone occupancy in all cell types, this highlights the importance of histone proteins as a control for genomic studies targeting histone post-translational modifications, as without normalizing to core histone levels, a change in a histone mark during aging could simply reflect a change in nucleosome occupancy at that particular genomic location (Chen et al., 2020).

### 2.2 Histone protein variants

Canonical histone proteins are assembled during replication, but histone variants, which are alternative histone proteins which vary slightly in sequence from their canonical counterparts, can be incorporated into DNA in a replication-independent manner (Henikoff, 2008). Of particular interest to the aging field is the histone H3 variant H3.3, which has four changes in amino acid sequence from the canonical H3 protein, and is enriched in transcriptionally active regions of DNA (Henikoff, 2008). Mammalian studies show that H3.3 increases during aging, with H3.3 taking over canonical H3 in the mouse by 18 months of age (Tvardovskiy et al., 2017). Analysis from human samples found H3.3 levels increased over the first decade of life in human brains (Maze et al., 2015), and increased in aged human fibroblasts, accompanied by a downregulation of H3.1 (Rogakou and Sekeri-Pataryas, 1999).

In *C. elegans*, HIS-71 and HIS-72 are H3.3-like proteins. Their mRNA and protein expression increases with age in adult *C. elegans* somatic cells, which are post-mitotic (Narayan et al., 2016; Piazzesi et al., 2016). Although loss of H3.3 through deletion of *his-71* and *his-72* did not impact the lifespan of WT worms, it did dramatically shorten the lifespan of multiple long-lived mutants, including the reduced insulin signaling *daf-2(e1370)* mutant, the germline-less *glp-1(bn18*) mutant, and the mitochondrial *nuo-6(qm200)* mutant (Piazzesi et al., 2016). This suggests that H3.3 may be necessary for survival in advanced age and may promote extended longevity in multiple pro-longevity contexts. It will be interesting in the future to test how HIS-71 and HIS-72 occupancy at chromatin changes during aging in WT worms and longevity mutants, and whether the genes at which H3.3 is incorporated during aging are changed in expression.

## 3 Nuclear architecture changes

### 3.1 Loss of heterochromatin

Domains within chromatin fibers can either be condensed, called heterochromatin regions, or relaxed, called euchromatin regions. Heterochromatin is typically associated with genes that are silenced, as the tightly wrapped DNA is difficult to access for transcription, while euchromatin is associated with active gene expression (Tamaru, 2010). The “loss of heterochromatin” model of aging, first put forth in 1997, proposes that a general decrease in heterochromatin occurs with age and leads to defects in gene silencing and age-related changes in gene expression (Villeponteau, 1997; Tsurumi and Li, 2012).

Bulk levels of heterochromatin-marking histone modifications H3K9me3 and H3K27me3 (Penagos-Puig and Furlan-Magaril, 2020) decline with aging in many model systems (Shumaker et al., 2006; Bracken et al., 2007; Maures et al., 2011; Djeghloul et al., 2016; Ito et al., 2018). This is particularly true in senescent cell cultures, which represent cells in a permanent state of cell-cycle arrest and dysfunction (López-Otín et al., 2013) and are a useful model to interrogate aging questions in long-lived species such as humans. Interestingly, genome-wide profiles of these heterochromatin histone marks using techniques like ChIP-seq are not always consistent with this hypothesis (See “Histone Post-translational Modifications” section below). Loss of transcriptional silencing has also been demonstrated during aging in multiple organisms, and is prominent in yeast aging (Smeal et al., 1996; Larson et al., 2012).

Some experimental evidence also supports a role for heterochromatin in promoting longevity, as a 50% reduction in the Heterochromatin Protein (HP1) in female *Drosophila* led to a drastically shorter lifespan than controls, whereas modest overexpression of HP1 increased lifespan (Larson et al., 2012). This was not the case in *C. elegans* however, where mutation or reduction by RNAi of one of the two *HP1* homologs, *hpl-2*, actually increased worm lifespan (Meister et al., 2011). Altering histone modifiers that target heterochromatin marks also affects lifespan in many organisms, however the lifespan phenotypes do not always follow the predictions of the “loss of heterochromatin” model of aging (See “H3K27me3” and “H3K9me3” sections below).

Somewhat paradoxically, along with a global loss of heterochromatin with aging, local regions of heterochromatin also accumulate in some human senescent cells (Narita et al., 2003). These are termed senescence-associated heterochromatin foci (SAHF), are accompanied by HP1, H3K9me3, and H3K27me3, and are typically thought to be transcriptionally repressed (Corpet and Stucki, 2014). Interestingly, Hi-C chromatin capture analysis, a technique that can detect regions of DNA that frequently contact each other (Lieberman-Aiden et al., 2009), revealed that even while SAHF form in senescent cells, the physical contacts within these regions of heterochromatin decrease, suggesting that SAHF may represent decondensed heterochromatin regions still marked by repressive histone marks and partially reconciling the appearance of SAHFs with the idea that heterochromatin is globally lost with aging (Chandra et al., 2015). In line with this, Tomimatsu et al. identified a subset of H3K9me3 peaks in a human fibroblast model of senescence that also contained the active mark H3K27ac and were identified as accessible regions as determined by assay for transposase-accessible chromatin with sequencing (ATAC-seq) (Tomimatsu et al., 2021). Some of these peaks showed gene activation, consistent with the idea that regions marked by H3K9me3 in senescent cells may represent a decondensed and more accessible form of chromatin than traditional heterochromatin (Tomimatsu et al., 2021).

Importantly, global reduction accompanied by local and region-specific gain of heterochromatin-associated histone modifications has also been seen in *C. elegans* somatic cells (Li et al., 2021) (See “H3K27me3” and “H3K9me3” sections below), suggesting that localized gain in heterochromatin marks may be a common feature of aging. These region-specific gains of heterochromatin highlight the importance of genome-wide and molecule-specific approaches coupled with gene expression analysis, and the subtlety required in interpreting global trends in chromatin during aging.

### 3.2 Nuclear morphology

Heterochromatin and euchromatin, besides correlating with different gene expression statuses, are also organized differently in the nucleus, with heterochromatin lining the nuclear periphery, and euchromatin residing preferentially in the nucleoplasm (Buchwalter et al., 2019) (See Figure 1). During aging, nuclear morphology is often seen to deteriorate (Tsurumi and Li, 2012), and the structure anchoring heterochromatin to the edge of the nucleus breaks down (Gruenbaum et al., 2002). The most notable example of such a breakdown occurs in the human premature aging disorder, Hutchinson-Gilford Progeria Syndrome (HGPS), which is typically caused by a germline mutation in lamin A that leads to an aberrant splice site in the gene and a protein product, termed progerin, that is missing part of the C-terminal region (De Sandre-Giovannoli et al., 2003; Eriksson et al., 2003). Lamin A, encoded by the human gene *LMNA*, is an essential component on the nuclear lamina, which provides structure for the nucleus by lining the nuclear periphery and anchoring heterochromatin (Dittmer and Misteli, 2011). Lamin A is normally modified by post-translational farnesylation, which may help target it to the nuclear membrane. It is later cleaved and the last 18 amino acid residues are removed, which also removes the farnesyl group from lamin A. Progerin lacks the residues in lamin A that are normally cleaved, and as a result, progerin is permanently farnesylated (Dittmer and Misteli, 2011). In HGPS, which shares some common features with natural aging, the heterochromatin marks H3K9me3 and H3K27me3 are lost over time and nuclear morphology breaks down (Goldman et al., 2004; Shumaker et al., 2006). Interestingly, low levels of progerin are also produced during natural human aging due to the use of a cryptic splicing site in *LMNA*, and nuclear morphology defects, similar to those seen in HGPS, are seen in cells derived from elderly individuals (Scaffidi and Misteli, 2006; Mosevitsky, 2022).

Nuclear morphology defects also occur during aging in *C. elegans*, characterized by folded nuclear lamina, blurred nuclear periphery, and intranuclear accumulation of the only lamin in *C. elegans*, LMN-1 (Haithcock et al., 2005). LMN-1 is required for a normal lifespan in the worm, as loss of function mutation or RNAi of *lmn-1* leads to a short lifespan. This is further supported by evidence that simultaneously reducing two interacting partners of LMN-1, EMR-1 and LEM-2, also shortens *C. elegans* lifespan (Haithcock et al., 2005). Some long-lived *C. elegans* strains show slower nuclear morphology defects during aging, including a dietary restriction mimic, *eat-2(ad1116)* (Charar et al., 2021) and the insulin receptor mutant *daf-2(e1370)* at 25°C (Haithcock et al., 2005; Zhao et al., 2017). This suggests that nuclear morphology could be correlated with lifespan.

However, evidence suggests that the nuclear morphology defects observed in *C. elegans* can be separated from the lifespan phenotype. Pérez-Jiménez et al. showed that while *daf-2(e1370)* mutants exhibited delayed nuclear morphology defects during aging at 25°C as previously reported, they exhibited a rate of nuclear morphology defects comparable to wildtype worms at 20°C, even while they remained very long-lived at 20°C (Pérez-Jiménez et al., 2014). Furthermore, Fan et al. demonstrated that nuclear blebs, local deformations of the nuclear envelope that form small protrusions and are characteristic of HGPS (Capell and Collins, 2006), increased with age in *C. elegans* but did not correlate with longevity (Fan et al., 2023). Specifically, interventions known to affect lifespan in *C. elegans*, such as aging at increased temperatures or long-lived *daf-2(e1370)* mutation at 20°C, did not consistently alter the rate of nuclear blebbing accumulation with aging.

Similarly, although blocking farnesylation and thereby preventing the permanent farnesylation of progerin is sufficient to extend the lifespan of mouse models of HGPS (Varela et al., 2008; Zhang et al., 2013), blocking farnesylation with three different pharmacological and genetic approaches in *C. elegans* was sufficient only to improve the age-related nuclear morphology defects, but not the lifespan, of wildtype worms (Bar et al., 2009; Bar and Gruenbaum, 2010). Although it is unknown whether *C. elegans* lamin is processed in the same way as human lamin A, and the interventions used to inhibit farnesylation were broad inhibitors rather than targeting LMN-1 farnesylation exclusively, the evidence taken together suggests that nuclear morphology defects and longevity can be uncoupled in *C. elegans*. This uncoupling has also been observed in a fish model of HGPS, which exhibits the nuclear morphology defects but not the shortened lifespan characteristic of HGPS (Tonoyama et al., 2018). It will be intriguing to uncover whether nuclear morphology and aging can be uncoupled in mammals. Further experimentation to understand how loss of *lmn-1* shortens lifespan of the worm and whether it does so by affecting nuclear morphology or by other mechanisms, will also be crucial for understanding the link between lamins, nuclear morphology, and aging.

## 4 Chromatin remodeling

Cells are presented with many occasions where DNA packaging into nucleosomes must be altered to facilitate a biological process. Nucleosomes must be assembled after replication, and re-positioned or ejected to alter the accessibility of regulatory regions and aid in DNA repair or transcriptional activation or repression (Clapier and Cairns, 2009). In order to accomplish this, cells utilize specialized chromatin remodelers, which use ATP hydrolysis to insert, move, or eject histones from chromatin. Many of the changes at chromatin we have discussed, such as loss of histones, changes in histone variants, and changes in chromatin accessibility, may be facilitated by chromatin remodelers. There are four classes of chromatin remodeling complexes, classified by the characteristics of the remodeler’s ATPase domain: SWI/SNF, ISWI, CHD, and INO80. There are many similarities as well as unique features among these remodelers (Clapier and Cairns, 2009), and many studies implicate these factors in aging and longevity processes, especially in *C. elegans*. Due to the abundance of experimental evidence in *C. elegans* and the availability of a recent review covering chromatin remodelers and aging (Swer and Sharma, 2021), we will focus here on evidence linking remodelers to longevity in the worm.

### 4.1 SWI/SNF family

The SWI/SNF (switching defective/sucrose nonfermenting) family of remodelers have been shown to work with the pro-longevity forkhead box O (FOXO) transcription factor, DAF-16, to control lifespan in *C. elegans*. Riedel et al. identified SWI/SNF components as a major cofactor in mediating DAF-16-dependent gene expression pathways (Riedel et al., 2013), which promote stress resistance and longevity and are required for the long lifespan of the reduced insulin signaling *daf-2* mutant (Kenyon et al., 1993; Murphy, 2006). They found that SWI/SNF chromatin remodelers of the BAF-subclass signature are recruited in part by DAF-16 to DAF-16-bound promoters, where they contribute to the activation of DAF-16-target genes. SWSN-1 and SWSN-4 were both required for the long lifespan of the *daf-2(e1370)* mutant in a DAF-16-dependent manner (Riedel et al., 2013). Bansal et al. had similar findings, and showed that SWI/SNF remodelers regulate the expression of the *daf-16d/f* isoform in adulthood, which contributes to the lifespan extension of the *daf-2(e1370)* mutant (Bansal et al., 2014).

Interestingly, moderate loss of *swsn-1* appears not to affect WT lifespan during aging at 25°C, but strong loss of function can decrease WT lifespan at 25°C (Riedel et al., 2013) and reduction of *swsn-1* by RNAi can decrease WT lifespan at 15°C (Zhou L. et al., 2019). Together, these observations suggest that SWI/SNF components cooperate with DAF-16 to modulate the expression of DAF-16d/f itself along with DAF-16 target genes. Interestingly, loss of SWSN-7, a signature member of the PBAF-subclass, which does not appear to work with DAF-16 (Riedel et al., 2013), also shortens *C. elegans* lifespan (Kuzmanov et al., 2014). Taken together with the evidence that a strong loss of function allele of *swsn-1* can shorten the lifespan of a *daf-16* null mutant (Riedel et al., 2013), this suggests that the SWI/SNF complex can modulate lifespan via DAF-16-dependent and independent mechanisms, which will be an interesting avenue for future investigation.

### 4.2 ISWI family

Members of the NURF (Nucleosome Remodeling Factor) complex, which belongs to the ISW1 (imitation switch) family of chromatin remodelers, have been implicated in longevity in *C. elegans*. However, the effects of the NURF complex components are variable and depend upon treatment and genetic background. Matilainen et al. showed that overexpression of ISW-1, the catalytic subunit of the NURF complex, extends lifespan of wildtype worms through controlling the expression of small heat-shock proteins (sHSPs) (Matilainen et al., 2017). ISW-1 is required for the full longevity of both a model of mitochondrial-mediated longevity, *cco-1* RNAi (Matilainen et al., 2017), and the reduced insulin signaling mutant, *daf-2(e1368* and *e1370)* (Curran et al., 2009). However, Matilainen et al. showed that knockdown of *isw-1* through RNAi is sufficient to shorten WT lifespan only when RNAi treatment is given to both the parental generation (during larval stages 1–3) and the test generation, suggesting that ISW-1 could promote longevity during a critical developmental window and in adulthood (Matilainen et al., 2017). Surprisingly, Müthel et al. demonstrated that *isw-1(n3294)* mutants, which are believed to represent a decrease in ISW-1 function (Andersen et al., 2006), are long-lived compared to WT worms (Müthel et al., 2019), and Guillermo et al. uncovered *isw-1* from a blind RNAi screen for chromatin factors that when knocked down increased longevity (Guillermo et al., 2021). This suggests that ISW-1 could have opposing roles in longevity in different contexts, and highlights the importance of testing multiple methods of reducing protein function in various conditions.

Evidence consistently suggests that NURF-1, a member of the NURF complex predicted to bind DNA and methylated histones, limits longevity, although again the results are context-dependent. Matilainen et al. found that knockdown of *nurf-1* extends WT lifespan (Matilainen et al., 2017), however the RNAi regimen again involved pre-treatment of the parental generation (from larval stages 1–3) and subsequent RNAi of the tested generation. Large et al. identified a 60-bp deletion in the *nurf-1* gene as being partially responsible for the differences in reproduction and longevity between two laboratory-adapted *C. elegans* strains, N2 and LSJ2, which originated from a single hermaphrodite and likely diverged due to laboratory maintenance conditions (Large et al., 2016). LSJ2, which contains the *nurf-1* deletion, was historically grown in nutrient-poor liquid culture conditions and has a longer natural lifespan at 25°C on plates compared to N2. When the *nurf-1* deletion was introduced into the N2 background, lifespan was increased, although not to the same extent as the LSJ2 strain. Importantly, the authors used a version of the N2 strain containing the ancestral alleles for *npr-1* and *glb-5*, which have been previously shown to have acquired variation in the commonly used N2 strain. They found when they compared the lifespan of the standardly used N2 strain with variants of *npr-1* and *glb-5* to a previously generated *nurf-1(n4295)* mutant, there was no change in longevity (Large et al., 2016). This is in contrast to the findings of Müthel et al., who demonstrated that the *nurf-1(n4295)* mutant is long-lived compared to the standard N2 strain (Müthel et al., 2019). The difference is puzzling given that both papers used the same allele of the *nurf-1* mutant (*n4295),* and that lifespan conditions were similar, with both papers aging worms at 25°C on plates containing the chemical 5-fluoro-2′-deoxyuridine (FUDR) to prevent progeny production. The only notable difference between the two protocols is that Müthel et al., who observed a long-lived phenotype, grew their worms at 15°C until larval stage 4 (L4), whereas Large et al., who observed no longevity phenotype, grew their worms at 20°C until the young adult stage, before both aging them at 25°C. Taken together, both members of the NURF complex seem to influence longevity in a context-specific manner in *C. elegans*.

### 4.3 CHD family

CHD (chromodomain, helicase, DNA binding) family members have also been implicated in longevity, but similarly to the ISWI family, the results are inconsistent throughout conditions and family members. The most notable complex formed by CHD family members is the NuRD (nucleosome remodeling and deacetylase) complex, which is unique because it contains both a nucleosome remodeler (either LET-418 or CHD-3 in *C. elegans* (Passannante et al., 2010)) and a histone deacetylase (Basta and Rauchman, 2015) (HDA-1 in *C. elegans* (Passannante et al., 2010)). The NuRD complex can thus act to alter the chromatin environment via both changing the position of nucleosomes and removing post-translational modifications of acetylation from histones (See “Histone Post-translational Modifications” section below). However, several members of the NuRD complex also belong to additional chromatin complexes and regulate diverse processes including development (Basta and Rauchman, 2015), complicating the interpretation of genetic results.

The core remodeling subunit of the NuRD complex in *C. elegans* can be either LET-418 or CHD-3. Müthel et al. identified that loss of CHD-3 by mutation (*chd-3(eh4*) mutant)) leads to a slightly extended lifespan in the worm (Müthel et al., 2019). While one report indicates that loss of LET-418 in the *let-418(n3536)* mutant leads to no impact on longevity (Golden et al., 2022), two others show that *let-418(n3536*) mutants are long-lived (De Vaux et al., 2013; Müthel et al., 2019). De Vaux et al. identified that the long lifespan of *let-418* mutants is partially dependent on DAF-16 and that LET-418 is required for the full extent of longevity of the *daf-2(e1370)* mutant, *age-1* RNAi worms, and *akt-1(RNAi);akt-2(ok939)* worms, all of which are factors in the insulin signaling pathway, suggesting that LET-418 is involved in this pathway (De Vaux et al., 2013). Importantly, while NuRD complex members do affect development, the *chd-3* mutant has been reported not to exhibit obvious developmental phenotypes, and the *let-418(n3536)* mutant is a temperature-sensitive mutant with normal germline and reproductive phenotypes when shifted to the restrictive temperature at the L4 stage (De Vaux et al., 2013).

The conflicting results regarding the lifespan of the *let-418(n3536)* mutant are reminiscent of those described above for the *nurf-1* mutant, as the only notable difference between the protocols is that De Vaux et al. and Müthel et al., both of whom observed a lifespan phenotype, developed their worms at 15°C until the L4 stage, while Golden et al., who did not observe a lifespan phenotype, developed their worms at 20°C until the L4 stage. Taken together, these results indicate that NuRD complex chromatin remodelers could negatively regulate lifespan, and that the precise developmental temperature used for growing worms could have a major impact on longevity phenotypes for members of chromatin remodeling complexes in *C. elegans*.

Reduction of NuRD complex component LIN-61 in the *lin-61(n3809)* mutant also led to a long lifespan at 25°C (Müthel et al., 2019), however, depletion of other NuRD complex members does not always lead to a long lifespan, and loss of several NuRD complex components leads to a decreased lifespan. Additionally, the effects of loss of certain components seem to be dependent on timing and temperature. Reduction of DCP-66 from the L4 stage by RNAi results in a decreased lifespan (Golden et al., 2022). Reduction of LIN-53 by RNAi initiated at the L4 stage led to no detectable lifespan phenotype (De Vaux et al., 2013), whereas *lin-53(n3368)* and *lin-53(n833)* mutants are short-lived compared to WT (De Vaux et al., 2013; Müthel et al., 2019; Zhu et al., 2020). Similarly, reduction of the histone deacetylase component of the NuRD complex, HDA-1, by RNAi initiated at the L4 stage or day 1 of adulthood at 25°C (De Vaux et al., 2013; Emerson et al., 2023) or in liquid culture initiated at the L1 stage at 20°C (Edwards et al., 2014) had no effect on lifespan, whereas worms aged on plates with RNAi initiated at the L1 stage exhibited a shorter lifespan than control worms at 20°C (Shao et al., 2020). While Müthel et al. found that *lin-40(ok905)* mutants exhibited no lifespan phenotype at 25°C (Müthel et al., 2019), Zhu et al. found *lin-40(yth27)* mutants to be significantly shorter lived than WT worms at 20°C, and found that overexpression of LIN-40 was sufficient to increase the lifespan of WT worms (Zhu et al., 2020).

NuRD complex components have also been demonstrated to be required for the mitochondrial unfolded protein response in *C. elegans*, and several components are required for the extended longevity of models of mitochondrial perturbation. Specifically, LIN-40 and LIN-53 are required for the long lifespan seen under *cco-1* RNAi conditions (Zhu et al., 2020), and HDA-1 is required for the long lifespan under *atp-2* RNAi conditions (Shao et al., 2020). In both cases, LIN-40 or HDA-1, respectively, were shown to be required for the normal change in RNA expression in response to mitochondrial perturbation (Shao et al., 2020; Zhu et al., 2020).

Apart from the NuRD complex, CHD-7, the only worm homolog to the class III CHD family, has been studied in lifespan regulation in *C. elegans* (Jofré et al., 2022). Mutants for *chd-7 (gk290* and *gk306*) are short-lived in *C. elegans*, however perplexingly, overexpression of CHD-7 with CHD-7::GFP also resulted in a shorter lifespan in WT worms, suggesting dosage of CHD-7 could be critical for lifespan regulation. The long lifespan of the *daf-2(e1370)* mutant is dependent on CHD-7, as the *chd-7(gk290)* mutant, which is dauer-defective, can shorten the lifespan of the *daf-2(e1370)* mutant to a similar extent as *daf-16(mu86)* mutation. The authors found that CHD-7 shares many chromatin binding sites with DAF-16, and also that CHD-7 works in the TGFβ signaling pathway, though whether these observations account for the lifespan phenotype of the mutant remain unclear (Jofré et al., 2022).

Altogether, members of the CHD family of chromatin remodelers have varied impacts on longevity in *C. elegans*, dependent on the protein, timing, and condition. It will be interesting for future studies to systematically identify how these factors impact lifespan under varied conditions, and how chromatin itself is altered in the mutants to affect longevity.

### 4.4 INO80 family

To our knowledge, there is no evidence connecting the INO80 (inositol requiring 80) family of chromatin remodelers to longevity in *C. elegans*. However, since INO80 has been implicated in telomere maintenance and senescence in mice (Min et al., 2013) and chronological lifespan in yeast (Garay et al., 2014), exploring INO80 components in *C. elegans* will be an interesting avenue for future study.

## 5 Histone post-translational modifications

Histone tails are frequently modified with post-translational modifications that often correlate with the expression of the genes they mark. Histone acetylation marks are typically associated with transcriptional activation, as the negative charge of the acetyl group added to the positive charge on lysine residues within histone tails neutralizes the charge and reduces the attraction between the histones and negatively charged DNA molecules, thus opening chromatin (Bannister and Kouzarides, 2011). As the addition of methyl groups does not universally impact the charge of the histone tail, methylation marks have properties more specific to the number of methyl groups added and location of methylation on the histone tail. Active methylation marks include trimethylation of lysine 4 on histone 3 (H3K4me3), which usually marks active promoters, and trimethylation of lysine 36 on histone 3 (H3K36me3), which marks the gene body of actively transcribed genes (Wang et al., 2009). Marks associated with transcriptional repression include trimethylation of lysine 27 on histone H3 (H3K27me3) and trimethylation of lysine 9 on histone 3 (H3K9me3), which mark heterochromatin (Penagos-Puig and Furlan-Magaril, 2020). Although there are many more histone marks present in the cell, those mentioned above are some of the best studied, especially in the context of longevity, and this review will focus on those. Additionally, because of the abundance of experimental evidence and because recent reviews have detailed changes in bulk histone modifications during aging (Booth and Brunet, 2016; Pal and Tyler, 2016; Denzel et al., 2019; Yi and Kim, 2020; Yu et al., 2020; Saul and Kosinsky, 2021), this review will focus on studies that examine genome-wide changes in histone modifications via chromatin profiling techniques, if available, and experimental evidence connecting histone writers, readers, and erasers to longevity, particularly in *C. elegans*.

### 5.1 Histone acetylation

Acetyl groups can be added to histone tails at many locations, and specific histone acetylation marks can be associated with different genomic features, for example H3K9ac typically marks promoter regions while H3K27ac is a mark of active enhancers (Wang et al., 2009; Zhang et al., 2015). However, as there are many histone acetylation marks and no known studies investigating the genome-wide changes in histone acetylation in *C. elegans*, we will focus this review on the factors that control histone acetylation, which have been implicated in longevity in *C. elegans* and many other model systems. The effects of reduction of either histone acetyltransferases (HATs), which place histone acetylation, or histone deacetylases (HDACs), which remove histone acetylation (Bannister and Kouzarides, 2011), on longevity are variable. Often, dramatically reducing HATs or HDACs will lead to a shortened lifespan, possibly because these factors are so ubiquitous in the cell and serve so many essential functions. However, HATs and HDACs can also serve roles to modulate longevity, often depending on the context. Therefore, there is no simple relationship between histone acetylation and longevity, but examining the factors involved can provide clues as to the complex relationship between acetylation and lifespan. In future experiments, it will be critical to uncover the genome-wide binding profiles of these factors in *C. elegans* during development, adulthood, and aging, to understand differences between each HAT/HDAC and why some, but not all, contribute to longevity.

#### 5.1.1 Histone acetyltransferases

Reducing the *C. elegans* HAT CBP/p300 homolog CBP-1 by RNAi initiated during development or in adulthood substantially shortened the lifespan of WT worms in two studies (Zhang et al., 2009; Zhou L. et al., 2019), while RNAi initiated at the L4 stage lengthened lifespan in another (Guillermo et al., 2021). Surprisingly, both Zhang et al. and Guillermo et al. utilized similar conditions, including aging worms at 20°C in the presence of FUDR, but obtained opposite results. CBP-1 has been shown to be required for lifespan extension by dietary restriction, *daf-2(e1370)* mutation (Zhang et al., 2009), and temperature-induced hormetic lifespan extension (Zhou L. et al., 2019) in *C. elegans*. Interestingly, knockdown of p300 in human cells has been shown to induce senescence (Prieur et al., 2011), suggesting that CBP/p300 could be a conserved pro-longevity factor in worms and humans in certain contexts. Mutation of another HAT gene, *hat-1*, can extend *C. elegans* lifespan (Müthel et al., 2019), suggesting it could limit longevity in worms.

#### 5.1.2 Histone deacetylases

HDACs are subdivided into classes based on their protein structure and cofactor required to remove acetylation. Class I, II, and IV HDACs require zinc as cofactors, while class III HDACs (sirtuins) require NAD^+^ as a cofactor (Park and Kim, 2020). While Class I HDACs are typically ubiquitously expressed, the others can be tissue-specific (Park and Kim, 2020). Classes I, II, and III have been studied in the context of lifespan in *C. elegans*, while it is unclear whether *C. elegans* possess Class IV HDACs (Milazzo et al., 2020).

##### 5.1.2.1 Class I and II HDACs

In *C. elegans*, the most well-studied Class I HDAC is HDA-1, which is part of both the NuRD complex and additional NuRD-independent HDAC complexes (See “CHD Family” in “Chromatin Remodeling” section above). As discussed above, loss of *hda-1* leads to either no change in lifespan (De Vaux et al., 2013; Edwards et al., 2014; Emerson et al., 2023) or a shortened lifespan in WT worms (Shao et al., 2020), depending on conditions. Interestingly, loss of SIN-3, a core component of another complex HDA-1 belongs to (SIN3/HDAC complex), also leads to a shortened lifespan in *C. elegans* (Pandey et al., 2018; Sharma et al., 2018; Müthel et al., 2019). Knockdown of SIN3 in *Drosophila* also shortens lifespan of both male and female flies (Barnes et al., 2014). Müthel et al. suggest that LIN-53, another member of the NuRD complex, also regulates lifespan through interaction with SIN-3 independently from NuRD (Müthel et al., 2019). Thus, in some contexts, the SIN3/HDAC complex may help to promote longevity.

The other two Class I HDACs in *C. elegans* (HDA-2 and HDA-3) have also been studied in longevity regulation. Reduction of HDA-2 or HDA-3 by RNAi has been shown to modestly extend lifespan, whereas constitutive knockout worms harboring mutations in *hda-2* (Edwards et al., 2014) or *hda-3* (Edwards et al., 2014; Kawamura and Maruyama, 2020) had shortened lifespans, suggesting that partial inhibition of HDACs could be beneficial for longevity, whereas full inhibition or inhibition during embryogenesis is detrimental for longevity (Edwards et al., 2014). A similar phenomenon has also been observed in *Drosophila*, where heterozygous loss of Rpd3, the HDAC1 homolog, leads to an extended lifespan (Rogina et al., 2002), but homozygous loss of Rpd3 is lethal (Mannervik and Levine, 1999). As HDACs are broad factors that are critical for development, it is perhaps not surprising that their effects can be highly variable. Along with the results suggesting that the lifespan phenotype caused by loss of *hda-1* is context-dependent in *C. elegans*, these studies underlie the importance of careful examination of experimental conditions, developmental timing, and partial versus complete gene depletion.

Similarly, mutation of Class II HDACs *hda-10* (Edwards et al., 2014) or *hda-4* (Edwards et al., 2014; Nikooei et al., 2020) have been shown to reduce WT lifespan in *C. elegans*. HDA-4 has also been shown to be required for the longevity of the *kin-29(oy38)* mutant (Nikooei et al., 2020). KIN-29, a salt-inducible kinase, inhibits HDA-4 by phosphorylation (van der Linden et al., 2007), suggesting that in WT worms and in the context of *kin-29* mutants, HDA-4 activity may promote longevity.

##### 5.1.2.2 Class III HDACs

The most notable example of an HDAC as a longevity factor is the Class III NAD^+^-dependent sirtuin, SIRT1 in mammals or SIR-2.1 in *C. elegans*, which is typically thought to promote lifespan. Sirtuins are deacetylases with both histone and non-histone targets, (Booth and Brunet, 2016) and SIRT1 has many important functions, including repressing repetitive elements and responding to DNA damage (Oberdoerffer et al., 2008). The role of SIRT1/SIR-2.1 in lifespan has been studied extensively and remains somewhat controversial due to conflicting results. *SIR2* was originally identified in yeast (Klar and Fogel, 1979) and was shown to impact lifespan in yeast in 1999, with deletion leading to a shortened lifespan, and overexpression of SIR2 leading to an extended replicative lifespan (Kaeberlein et al., 1999). Knockout of SIRT1 in mice does lead to a shortened lifespan, but SIRT1 knockout mice often die in the perinatal period and exhibit developmental defects (Herranz and Serrano, 2010). Overexpression of SIRT1 in whole mice promotes healthy aging, but fails to extend lifespan (Herranz et al., 2010), whereas overexpression in just the mouse brain has been shown to increase lifespan (Satoh et al., 2013).

Overexpression of SIR2 homologs in worms and flies, SIR-2.1 and dSir2, were both shown to increase lifespan (Tissenbaum and Guarente, 2001; Rogina and Helfand, 2004; Viswanathan et al., 2005). In *C. elegans*, the long-lifespan caused by overexpression of SIR-2.1 is dependent on DAF-16 (Tissenbaum and Guarente, 2001), and evidence suggests that under stress conditions, SIR-2.1 can activate DAF-16 via interaction with 14-3-3 proteins, which are also required for the lifespan extension of SIR-2.1 overexpression (Berdichevsky et al., 2006). This activation appears to occur indirectly through repression of the chromatin adaptor protein HCF-1, which is bound and repressed by SIR-2.1 and then subsequently binds 14-3-3 proteins and inhibits DAF-16 from reaching its target promoters (Rizki et al., 2011).

A follow-up report suggested that the lifespan-extending effects of SIR-2.1 overexpression in worms and flies was due to genetic background rather than overexpression of SIR-2.1 (Burnett et al., 2011), however subsequent outcrossing demonstrated that overexpressing SIR-2.1 still increased lifespan in *C. elegans*, but perhaps to a lesser extent than originally reported (Viswanathan and Guarente, 2011). Further analysis identified that the long lifespan caused by overexpression of SIR-2.1 was dependent on the absence of a common background mutation which was identified in the strains of the Burnett et al. paper (Zhao et al., 2019). Specifically, Zhao et al. identified a common background mutation in the gene *fln-2* in a commonly distributed WT stock (termed *fln-2(ot611)*) that increased lifespan compared to the wildtype *fln-2* allele and abrogated the effect of SIR-2.1 overexpression on lifespan. Interestingly, the authors also determined that the increased lifespan of SIR-2.1 overexpression was dependent on the presence of the chemical FUDR (Zhao et al., 2019), which is commonly used to prevent progeny production in the worm, but which can also have additional effects on worm physiology and can interact with certain lifespan-modulating pathways (Aitlhadj and Stürzenbaum, 2010; Van Raamsdonk and Hekimi, 2011; Rooney et al., 2014; Wang et al., 2019). Taken together, SIRT1/SIR-2.1 is one of the best studied factors to extend lifespan and healthspan in organisms from yeast to mice, and though the effects of overexpression seem to be context-dependent, evidence consistently suggests that SIRT1/SIR-2.1 promotes healthy aging and longevity.

### 5.2 H3K4me3

#### 5.2.1 Age-associated changes

**FIGURE 2:**
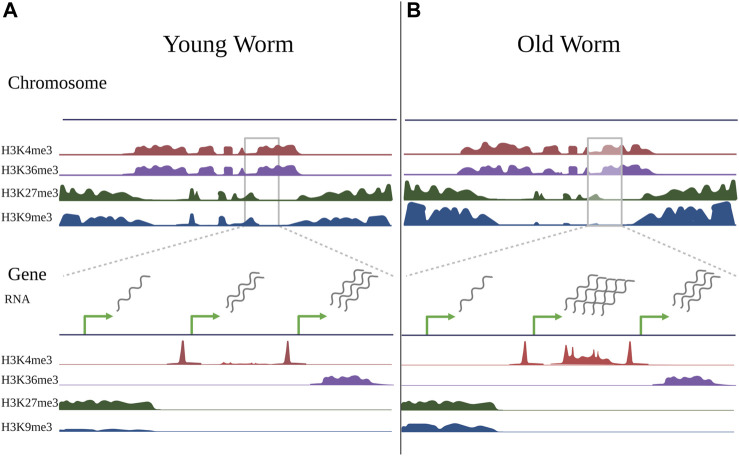
Changes in histone modifications during aging. Schematic illustrating changes in histone modifications observed during aging in somatic cells of the worm *C. elegans*, compiled from ChIP-seq experiments of germline-less *glp-1(e2141)* mutants in young worms (day 2 adults) vs old worms (day 12 adults) (Pu et al., 2015; Pu et al., 2018; Li et al., 2021). Some changes are evident at a chromosome-wide view (top), but become more evident when viewing smaller gene-specific regions (bottom). When viewing histone marks from a chromosome-wide view (top), active histone marks (H3K4me3 and H3K36me3) typically occupy similar regions of chromatin, as do repressive marks (H3K27me3 and H3K9me3) in *C. elegans*, however the regions occupied by active and repressive marks are distinct. Compared to young worms **(A)**, the genome-wide distribution of histone marks in old worms **(B)** is typically relatively stable, with local changes that both increase and decrease in direction. With aging, repressive marks, particularly H3K9me3, show an overall decrease, however this is region-specific, and the marks tend to decrease in the chromosome center and increase in the chromosome arms. There may also be somewhat of an invasion of active marks into domains normally occupied by repressive marks. When viewing individual peaks (bottom), H3K4me3, which typically marks promoter regions, exhibits age-related changes in gene bodies that typically correlate with gene expression changes. H3K36me3 is relatively stable with age, and genes marked by H3K36me3 tend to be stable in expression during aging. Most H3K27me3- and H3K9me3-marked regions do not change with age, but many H3K9me3-marked regions exhibit local gains with age, although these changes are not well-correlated with gene expression changes. Green arrows represent transcription start sites of theoretical genes. Note that the theoretical genes shown here represent separate examples of changes observed in histone modifications during aging and do not represent a real instance of genes in immediate proximity.

In *C. elegans*, Pu et al. profiled H3K4me3 during development (larval stage 3), adulthood (adult day 2), and old age (adult day 12) in somatic cells using germline-less *glp-1(e2141)* mutants. They found that most H3K4me3 levels were established during development and did not display age-related changes (Pu et al., 2018). Importantly, 30% of H3K4me3-marked regions did change during aging, and this 30% tended to be regions within gene bodies which were not marked by H3K4me3 in development, but which gained the mark in adulthood (See Figure 2). Of the peaks that changed with age, approximately 45% increased (1,168 peaks), and 55% decreased (1,400 peaks), and these changes correlated with gene expression changes during aging as expected (Pu et al., 2018). This is consistent with site-specific changes in H3K4me3 rather than global trends.

This gain of H3K4me3 in non-canonical regions is also seen in yeast. Cruz et al. found that H3K4me3 levels at promoter regions increased with age, gaining marks on promoters that were not typically marked with H3K4me3 at a young age. This increase in H3K4me3 was highly correlated with genes that increased in mRNA expression with age (Cruz et al., 2018). Of note, Cruz et al. limited their analysis to promoter regions, and the overall induction of H3K4me3 they saw is consistent with the genome-wide induction of transcription that occurs with age in yeast (Hu et al., 2014).

In human senescent cells, Shah et al. identified that H3K4me3 forms broad domains spanning hundreds of kilobases, as opposed to the sharp peaks typically seen for H3K4me3 in healthy cells (Shah et al., 2013). A similar, though less dramatic phenomenon, was observed in aged murine HSCs, where over half of H3K4me3-marked regions broadened in aged cells, while fewer than 10% of H3K4me3-marked regions shrank (Sun et al., 2014). These changes were again correlated with gene expression changes during aging. Other studies observed more modest changes. McCauley et al. noted that, while the overall distribution of H3K4me3 did not change with age in human mesenchymal stem cells (hMSCs), there was a slight increase in H3K4me3 enrichment in promoters, consistent with a slight opening of chromatin (McCauley et al., 2021). Liu et al. found that H3K4me3 distribution remained constant in quiescent stem cells isolated from muscles of old versus young mice, with a slight decrease in signal strength at transcription start sites (TSSs) (Liu L. et al., 2013). Similarly, Wood et al. found that H3K4me3 distribution did not change in aged *Drosophila* heads, but the average H3K4me3 enrichment decreased (Wood et al., 2010).

Across model systems, H3K4me3 responds to aging, often increasing in a region-specific manner, and changes are typically well correlated with age-related changes in gene expression. Interestingly, regions bound by H3K4me3 that change during aging are frequently non-canonical in some way, either representing genes or regions of genes not commonly bound by H3K4me3, or peaks shaped differently from those normally seen in H3K4me3-bound regions.

#### 5.2.2 Factors linked to longevity

Many factors that bind or modify H3K4me3 have been linked to longevity, especially in *C. elegans*. The COMPASS complex, which places H3K4me3, has in particular been an area of active study. A seminal paper from Greer et al. showed that reduction of *C. elegans* COMPASS complex components, SET-2, ASH-2, and WDR-5, through mutation or RNAi, extends worm lifespan (Greer et al., 2010)*,* suggesting that H3K4me3 could limit lifespan*.* Strikingly, this longevity could be inherited to WT offspring with functional SET-2, ASH-2, and WDR-5 for up to four generations (Greer et al., 2011). This was further supported by evidence that reduction of the H3K4me3 demethylase, RBR-2, increased H3K4me3 and reduced worm lifespan (Greer et al., 2010; Maures et al., 2011), and that RBR-2 is required for the inheritance of longevity in WT offspring COMPASS complex mutants (Greer et al., 2011). Demethylase-deficient mutants of LID, the *Drosophila* homolog of RBR-2, also exhibit reduced lifespan in male flies (Li et al., 2010), suggesting this pattern could be somewhat conserved. Strikingly, the longevity results of the COMPASS complex mutants oppose those found in yeast, where COMPASS complex components are required for normal replicative lifespan and expression of genes during aging (Cruz et al., 2018).

The results in worms have also varied between laboratory and conditions, suggesting that the effects of the COMPASS complex are highly dependent on the environment. Whereas reduction of RBR-2 was originally shown to shorten lifespan (Greer et al., 2010; Maures et al., 2011), RNAi of *rbr-2* can actually extend the lifespan of WT worms aged with the chemical FUDR (Lee et al., 2003; Greer et al., 2010; Ni et al., 2012), and results conflict on whether loss of *rbr-2* can extend lifespan in a germline-less *glp-1(e2141)* mutant background (Greer et al., 2010; Ni et al., 2012). In fact, Alvares et al. reported that *rbr-2* mutants were slightly long-lived at both 20°C and 25°C in the absence of FUDR (Alvares et al., 2014), further confounding the results.

Several papers have also reported failure to reproduce the longevity phenotypes of COMPASS complex mutants, with Caron et al. reporting that two different alleles of the *set-2* mutant, including the *set-2(ok952)* mutant utilized by Greer et al., were actually short-lived compared to WT worms (Caron et al., 2022). Lee et al. reported that the long lifespan caused by reduction of WDR-5 (with RNAi or in the *wdr-5(ok1417)* mutant) was only present after maintaining worms with lowered WDR-5 for many generations (Lee et al., 2019). To reconcile these differences, Silva-García and Mair created two new *set-2* mutants using CRISPR/Cas9 genome-editing and observed that loss of *set-2* does extend *C. elegans* lifespan, but the effects vary based on food source (Silva-García and Mair, 2022). In the lab, *C. elegans* consume bacteria, and two of the most popular strains of bacteria used to feed worms are the *Escherichia coli* strains OP50 and HT115. OP50 is frequently used for standard maintenance, while HT115 is usually used for RNAi experiments. The metabolites available to *C. elegans* differ between these two food sources, and this has been shown to affect various phenotypes in the worm, including stress response and longevity in certain conditions (Zhou J.-J. et al., 2019; Revtovich et al., 2019; Stuhr and Curran, 2020). Silva-García and Mair found that loss of *set-2* extended *C. elegans* lifespan when aged on HT115, but not OP50 bacteria (Silva-García and Mair, 2022), which may partially account for the discrepancies seen in the field.

The mechanism of lifespan extension for COMPASS complex mutants and the mechanism of the inheritance of this longevity has also been an active area of study. Han et al. showed that long-lived COMPASS complex mutants on HT115 bacteria have high levels of lipid accumulation, and factors required for maintaining high lipid levels were also required for the lifespan extension of COMPASS mutants (Han et al., 2017). Interestingly, Wan et al. showed that WDR-5, but not SET-2 or ASH-2, was required for the inheritance of fat accumulation in a *C. elegans* model of obesity (Wan et al., 2022), raising the question of whether inheritance of fat accumulation could contribute to the inheritance of longevity in COMPASS mutants. Importantly, Greer et al. found that, while the longevity was inherited in WT offspring of COMPASS complex mutants, the bulk level decrease in H3K4me3 that is seen in COMPASS complex mutants is not inherited to WT offspring (Greer et al., 2011), presumably due to the now-functional COMPASS complex components themselves. Therefore, the question of what epigenetic signal could mediate the longevity in WT offspring remained puzzling. Lee et al. suggest that the signal is H3K9me2, which they found to be positively correlated with longevity (Lee et al., 2019). They found that the H3K9me2 methyltransferase MET-2 was required for the inheritance of longevity in *wdr-5* mutants, and that mutation of the putative H3K9me2 demethylase, JHDM-1, resulted in a long lifespan which was similarly inherited to WT offspring for several generations. Importantly, they identified that a subset of genes marked by high levels of H3K9me2 in long-lived *jhdm-1* mutants also showed high H3K9me2 in long-lived WT offspring of *jhdm-1* mutants, suggesting H3K9me2 could be a heritable signal for longevity (Lee et al., 2019).

Apart from the COMPASS complex mutants, the H3K4me3 reader, SET-26, has been shown to limit longevity in *C. elegans* (Hamilton et al., 2005; Greer et al., 2010; Ni et al., 2012). SET-26 does not directly add or remove H3K4me3, but rather binds to the mark and could contribute to regulation of the chromatin environment through recruitment of additional chromatin factors. In contrast to the COMPASS complex mutants, the longevity caused by SET-26 inactivation is not heritable to WT offspring (Greer et al., 2011). Similarly, while COMPASS complex proteins are believed to modulate longevity through the germline (Greer et al., 2010), SET-26 acts in somatic cells to limit lifespan, and the paralog of SET-26, called SET-9, which is expressed exclusively in the germline, does not modulate lifespan (Wang et al., 2018). Interestingly, SET-26 may modulate longevity in part by recruiting HCF-1, the chromatin adaptor protein shown to repress DAF-16, to chromatin in somatic cells (Emerson et al., 2023). Inactivation of either *set-26* or *hcf-1* increases worm lifespan, and the factors act in the same genetic pathway to modulate longevity. The two proteins share common binding sites at chromatin, regulate common sets of genes, and both require the histone deacetylase HDA-1 (Emerson et al., 2023) and the transcription factor DAF-16 (Li et al., 2008; Ni et al., 2012) for their longevity. Interestingly, SET-26 can only competently bind H3K4me3 marks when they are flanked by nearby acetylation (Wang et al., 2018), so the genetic interaction between SET-26, HCF-1, and HDA-1 is particularly interesting and warrants further investigation.

Altogether, perturbation of H3K4me3 readers, writers, and erasers is strongly linked to longevity in *C. elegans*, but whether altering H3K4me3 is beneficial or detrimental to longevity depends on the experimental conditions and factors tested.

### 5.3 H3K36me3

#### 5.3.1 Age-associated changes

Using ChIP-seq profiling in young and aged germline-less *glp-1(e2141)* worms, Pu et al. found that H3K36me3 remained largely stable during worm aging, and a small number of sites increased and decreased with age (Pu et al., 2015). While the H3K36me3 marks themselves tended not to change, Pu et al. identified that genes that dynamically changed in expression during aging were either unmarked or marked by low levels of H3K36me3 in their gene bodies, implicating H3K36me3 as an important mechanism to prevent mRNA changes during aging (Pu et al., 2015) (See Figure 2). The anti-correlation between H3K36me3 and RNA changes during aging seems to be well-conserved in diverse organisms. Although global H3K36me3 enrichment decreases with age in *Drosophila* heads (Wood et al., 2010), the observation that genes that change with age tend to be marked by low levels of H3K36me3 in their gene bodies remained true (Pu et al., 2015). Sen et al. found that H3K36me3 levels measured by ChIP-seq were also largely stable during yeast aging, but decreased on the gene bodies of 244 genes that were reproducibly identified to exhibit cryptic transcription (identified through intragenic transcripts) with age (Sen et al., 2015). This pattern holds true even in human cells, where McCauley et al. identified an increase in cryptic transcription accompanied by a decrease in H3K36me3 marks on gene bodies in aged hMSCs (McCauley et al., 2021). These highly consistent studies implicate H3K36me3 as a potential mechanism to prevent aberrant transcriptional programs during aging.

#### 5.3.2 Factors linked to longevity

Like the genome-wide profiling data, the evidence linking factors associated with H3K36me3 to longevity are the most consistent of any histone mark presented here. The data indicate that loss of H3K36me3 is detrimental for longevity, as would be expected if H3K36me3 were a mechanism to prevent aberrant RNA expression in old age. Reduction of the *C. elegans* H3K36me3 methyltransferase, MET-1, by mutation or RNAi, reduces bulk levels of H3K36me3 and shortens the lifespan of both WT and germline-less *glp-1(e2141)* mutants (Pu et al., 2015). The same is true in yeast, where loss of MET-1 homolog, Set2, leads to a decreased replicative lifespan (Sen et al., 2015). Conversely, loss of the H3K36me3 demethylase, JMJD-2, increases lifespan in WT worms, although puzzlingly RNAi of *jmjd-2* has no effect on the lifespan of *glp-1(e2141)* mutants (Ni et al., 2012). This discrepancy could be caused in part due to JMJD-2’s joint putative role as a demethylase for H3K36me3 and H3K9me3 (Whetstine et al., 2006). Loss of the yeast H3K36me2/me3 demethylase, Rph1, also extends replicative lifespan (Sen et al., 2015), consistent with a model by which H3K36me3 promotes longevity, possibly by preventing aberrant RNA expression during aging.

### 5.4 H3K27me3

#### 5.4.1 Age-associated changes

In *C. elegans*, the genome-wide distribution of H3K27me3 stays largely stable with age. Li et al. compared H3K27me3 in old (day 12 adult) versus young (day 2 adult) germline-less *glp-1(e2141)* worms using ChIP-seq and found that while overall H3K27me3 signal slightly decreased in aged worms, very few regions (1.1%) reached statistical significance (Li et al., 2021) (See Figure 2). Of the few regions that reached significance, the majority (59 of 66) showed increased H3K27me3 marks in aged worms, and the genes overlapping with these regions tended to decrease in expression during aging (Li et al., 2021). Interestingly, Maures et al. found that global H3K27me3 levels measured by western blot analysis dropped precipitously by day 14 in *glp-1(e2141)* mutants (Maures et al., 2011), and it will be interesting in the future to determine whether the distribution of H3K27me3 peaks changes at extreme old age (day 14 and beyond) in these worms.

In aged murine HSCs, Sun et al. also found that H3K27me3 peak number did not change dramatically with age, but individual peaks did reproducibly change (Sun et al., 2014). Like in worms, the majority of sites that were identified as significantly differentially bound by H3K27me3 in aged cells increased (402 of 526). The overall signal length of H3K27me3 increased, as did the signal at the transcription start site (TSS), which increased by around 50% in old cells (Sun et al., 2014). Liu et al. also found a general increase in H3K27me3 in quiescent stem cells isolated from muscle of old versus young mice (Liu L. et al., 2013). H3K27me3 increased in both intergenic regions and at TSSs with age, but the correlation between gene expression change and altered H3K27me3 with age was low (Liu L. et al., 2013).

In human senescent cells, Shah et al. identified widespread changes in H3K27me3, with similar numbers of large regions exhibiting increased or decreased H3K27me3 signal (Shah et al., 2013). In contrast, Chandra et al. identified very few global changes in H3K27me3 in human senescent cells (Chandra et al., 2012). Interestingly, both papers used the same cell line (IMR90 human fibroblasts), but Shah et al. induced senescence via replicative senescence (continual passaging until cell cycle arrest), while Chandra et al. used oncogene-induced senescence (activation of an oncogene leading to cell cycle arrest). Thus, although bulk levels of H3K27me3 have been shown to decrease with aging or senescence in several systems (Shumaker et al., 2006; Bracken et al., 2007; Maures et al., 2011; Ito et al., 2018), genome-wide analysis often identifies local regions of H3K27me3 that increase in aged cells, and the patterns identified depend on the cell type and model system used.

#### 5.4.2 Factors linked to longevity

Alteration of factors that modify H3K27me3 has been extensively linked to longevity in *C. elegans*, however the evidence has been made somewhat confusing by the observation that mutations that either increase or decrease H3K27me3 can extend lifespan. Specifically, loss of components of the Polycomb Repressive Complex (PRC2), MES-2, MES-3, and MES-6, which places H3K27me3, extends lifespan in *C. elegans* (Ni et al., 2012; Guillermo et al., 2021). This would indicate that reducing H3K27me3 could be beneficial for lifespan. However, reduction of the putative H3K27me3 demethylases UTX-1 and JMJD-3.2, which should increase H3K27me3 levels, also extends lifespan in *C. elegans* (Jin et al., 2011; Maures et al., 2011; Ni et al., 2012; Guillermo et al., 2021), although complete homozygous loss of UTX-1 is lethal (Kemphues et al., 1988). The lifespan extension caused by reduction of UTX-1, JMJD-3.2, and MES-2 are all dependent on DAF-16 (Jin et al., 2011; Maures et al., 2011; Guillermo et al., 2021). Interestingly, the *daf-2* gene is a direct H3K27me3 demethylase target of UTX-1, and *daf-2* RNA expression depends on UTX-1 (Jin et al., 2011). As UTX-1 requires its demethylase domain to modulate lifespan (Guillermo et al., 2021), this could be consistent with a role of UTX-1 in directly regulating H3K27me3 levels on *daf-2* to modulate lifespan (Jin et al., 2011).

Consistent with the observation that altering both H3K27me3 methyltransferases and demethylases increases lifespan, overexpressing either JMJD-3.2 or UTX-1 also extends *C. elegans* lifespan (Guillermo et al., 2021). Interestingly, Guillermo et al. identified that longevity mediated by knockdown of UTX-1 occurred through its actions in epidermal cells, neurons, and intestinal cells, whereas longevity mediated by overexpression of UTX-1 occurred through its actions in neurons and intestinal cells, but not epidermal cells (Guillermo et al., 2021). While knockdown of the JMJD-3.2 paralog, JMJD-3.1, does not lead to longevity in WT worms (Guillermo et al., 2021), overexpression of JMJD-3.1 can extend WT lifespan (Labbadia and Morimoto, 2015; Merkwirth et al., 2016) and induce the mitochondrial unfolded protein response (Merkwirth et al., 2016). JMJD-3.1 is also required for longevity induced by mitochondrial dysfunction (Merkwirth et al., 2016), suggesting that JMJD-3.1 may also have a role in longevity in specific situations.

Altering H3K27me3 levels is likely to contribute to the modulation of longevity in *C. elegans*, but there is no simple relationship between H3K27me3 and longevity. Understanding the tissue specific genome-wide targets of each H3K27me3 methyltransferase and demethylase under basal and overexpression conditions may help to further untangle the relationship between each factor, H3K27me3, and longevity. In *Drosophila*, heterozygous mutations in PRC2 components, E(Z) and ESC, decrease H3K27me3 and extend longevity. The extension requires TRX, an antagonist of the PRC2 complex (Siebold et al., 2010). Thus, examples from both *C. elegans* and *Drosophila* challenge the “loss of heterochromatin” model of aging, which would predict that mutations that decrease H3K27me3 should decrease longevity. However, Ito et al. demonstrated that reduction of the PRC2 H3K27me3 methyltransferase component, EZH2, in human fibroblasts induces senescence through both H3K27me3 independent and dependent mechanisms (Ito et al., 2018), supporting the “loss of heterochromatin” model and highlighting the variable roles of methyltransferases in different contexts and organisms.

### 5.5 H3K9me3

#### 5.5.1 Age-associated changes

Along with profiling H3K27me3, Li et al. also profiled changes in H3K9me3 that occur with aging in germline-less worms. They found that the overall H3K9me3 signal decreased in H3K9me3-marked regions during aging (Li et al., 2021). Interestingly, when they examined the boundaries of H3K9me3-marked regions in young and old worms, they noticed a deterioration of strong boundaries between active and repressive chromatin marks. Specifically, when they examined ChIP-seq data profiling H3K4me3 and H3K36me3, they found that these active marks showed a steep drop-off in signal at the beginning of H3K9me3 peak regions, consistent with the idea that active and repressive histone marks often occupy mutually exclusive domains. However, in old worms, they noticed a degradation of this drop-off. There were higher levels of H3K4me3 and H3K36me3 in regions marked by H3K9me3, suggesting an invasion of active marks into repressive regions of chromatin in old worms (Li et al., 2021). However, whether this invasion occurs within a single cell type, (i.e., co-occurrence of active and repressive marks in the same regions), or in different cell types (i.e., active marks invade regions in one cell type that are occupied by repressive marks in another cell type but do not co-occur in the same cell) is unclear given the study was conducted with whole worms. Similar to the H3K27me3 data, Li et al. found that when they asked for regions that were statistically significantly different in H3K9me3 between young and old worms, the majority of peaks (95%) did not observably change with age. Of the 595 peaks that did change with aging, 97% exhibited increased H3K9me3 marks with age (Li et al., 2021). Thus, while the overall levels of H3K9me3 declined, the region-specific changes that are most reproducible and reach statistical significance actually increased (See Figure 2). However, the H3K9me3 peaks that significantly changed during aging were not well-correlated to genes that changed in expression during aging (Li et al., 2021), thus the functional consequence of these changes is unclear.

Similarly, Chandra et al. found that global patterns of H3K9me3 did not change during oncogene-induced senescence in human cells, and the limited regions that did change were not well-correlated with gene expression changes (Chandra et al., 2012). Price et al. found that, although absolute levels of H3K9me3 increased in aged mouse livers as determined by mass spectrometry, ChIP-seq of H3K9me3 in old and young mouse livers resulted in similar numbers of called peaks (Price et al., 2020). Strikingly, only about 29% of peaks identified in old mouse livers overlapped with peaks identified in young livers, suggesting the distribution of H3K9me3 changed dramatically with age, with a number of peaks both gained and lost (Price et al., 2020). Park and Belden also found that H3K9me3-bound regions exhibited both significant increases and decreases in old versus young zebrafish (Park and Belden, 2018), whereas Wood et al. found that the normal pattern of H3K9me3 enrichment on *Drosophila* chromosomes was lost during aging in the fly brain (Wood et al., 2010). Overall, changes to H3K9me3 during aging seem to depend on the model system used. While loss of H3K9me3 is considered a hallmark of aging in senescent cells (Shumaker et al., 2006; Djeghloul et al., 2016), region-specific enrichment of H3K9me3 can be seen in many organisms, but the functional consequence of these changes will require further investigation.

#### 5.5.2 Factors linked to longevity

Several putative methyltransferases for H3K9me3 have been linked to longevity in *C. elegans*. SET-25 and SET-32 are putative H3K9me3 methyltransferases that have been studied for their roles in establishing transgenerational epigenetic silencing in the worm (Woodhouse et al., 2018), and both factors have been implicated in lifespan. Loss of SET-32 has been shown to extend WT lifespan, either by mutation or RNAi on plates with or without FUDR (Greer et al., 2010; Ni et al., 2012; Woodhouse et al., 2018). However, the precise role for SET-32 in regulating H3K9me3 is unclear. Although SET-32 has been shown to be required for H3K9me3 placement at specific loci in response to RNAi (Spracklin et al., 2017), Woodhouse et al. found that two different alleles of *set-32* mutants did not show global defects in H3K9me3 in proteomics (Woodhouse et al., 2018), suggesting that SET-32 could regulate H3K9me3 in a context-dependent manner. Loss of SET-25 does cause global reductions in H3K9me3 consistent with the role of a major methyltransferase (Woodhouse et al., 2018), however its loss only causes an extension of lifespan when worms are aged in the presence of FUDR (Ni et al., 2012) and not in its absence (Greer et al., 2010; Woodhouse et al., 2018).

Another putative H3K9me methyltransferase, SET-6, can mediate methylation of H3K9me2 and H3K9me3 (Yuan et al., 2020). Yuan et al. found that SET-6 operates in the nervous system of *C. elegans* to regulate healthy aging, and that loss of SET-6 by mutation in the *set-6(ok2195)* mutant increased levels of 5-HT and dopamine, ameliorated age-related behavioral changes, and increased lifespan. This lifespan extension occurred independently from DAF-16, but in the same pathway as dietary restriction (mimicked by *eat-2* mutation) and mitochondrial dysfunction (induced by *clk-1* mutation) (Yuan et al., 2020). This is in line with findings from Huang et al., who found that deletion of *set-6* or *set-32* extends lifespan in WT worms and synergistically increases lifespan of *daf-2(e1370)* mutants (Huang et al., 2022), consistent with SET-6 and SET-32 operating in a separate longevity pathway from DAF-2/DAF-16. However, *set-6* was also uncovered from two separate RNAi screens as a target that decreased WT lifespan in *C. elegans* (Greer et al., 2010; Ni et al., 2012), raising the question of whether dosage of *set-6* reduction is important for longevity, or if other factors such as compensation in the perpetual mutant could contribute to the differences seen by *set-6(ok2195)* mutants and *set-6* RNAi.

Altogether, loss of H3K9me3 methyltransferases often increases lifespan in *C. elegans*, suggesting a role for H3K9me3 in limiting lifespan. However, these results must be taken with caution as loss of SET-25 and SET-6 do not consistently alter lifespan in the worm, while SET-32 alters H3K9me3 only in certain situations.

## 6 DNA methylation

DNA itself can also be covalently modified by the addition of methyl groups, with the most commonly studied modification being the methylation of cytosine at the 5th position carbon (5-mC). 5-mC, a mark associated with gene silencing, has been extensively studied during aging, with a number of highly reproducible changes identified in human aging believed to represent an “aging clock” (Horvath, 2013; Horvath, 2015). Generally, 5-mC is thought to globally decrease during aging, with local increases at promoters, consistent with a model of general de-repression at chromatin with local repression of certain genes (Wang K. et al., 2022). However, when considering several recent studies that looked at CpG changes during aging at a large scale in humans and mice, Unnikrishnan et al. noted only a small fraction of 5-mC modifications at CpG sites (typically <5%) were changed during aging, and of those that did change, the majority of studies saw more hypermethylation than hypomethylation during aging (Unnikrishnan et al., 2019). Thus, more genome-wide studies will be needed to resolve precise changes in DNA methylation throughout aging.

As *C. elegans* are not believed to possess substantial levels of 5-mC (Wenzel et al., 2011), and 5-mC changes during aging have been reviewed elsewhere (Jung and Pfeifer, 2015; Bell et al., 2019; Wang K. et al., 2022), we will not go into further detail in this review. *C. elegans* have recently been identified to possess detectable levels of a different form of DNA methylation, methylation of adenosine at the 6th position NH_2_ group (6-mA) (Greer et al., 2015), and this modification has already been implicated in transgenerational longevity in the worm (Greer et al., 2016; Wan et al., 2021), making DNA methylation a promising avenue for future longevity research in the worm.

## 7 Discussion

**FIGURE 3:**
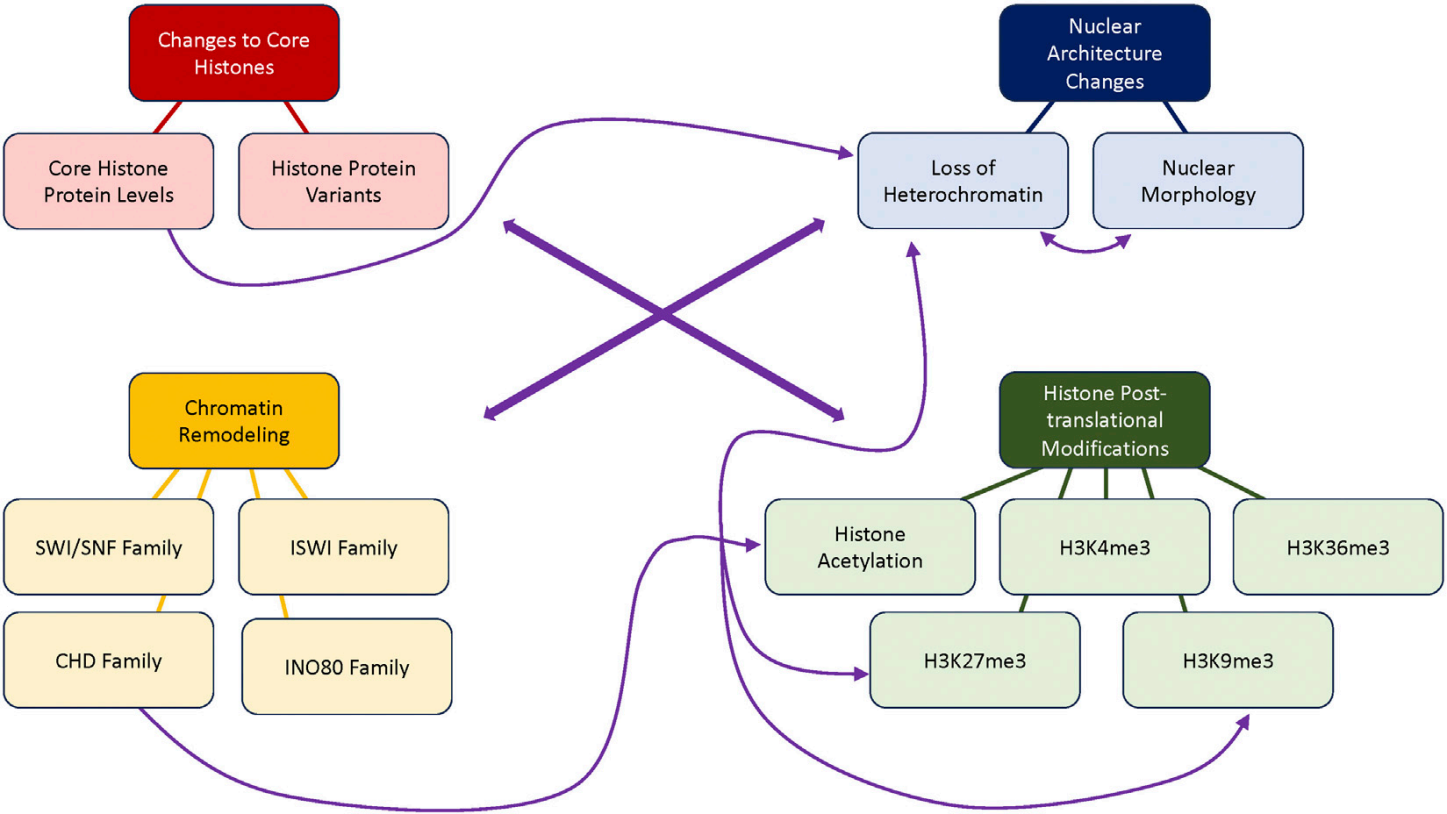
Connections between age-related changes in chromatin. Summary of topics (darkly colored) and sub-topics (lightly colored) covered in this review and the potential connections between those topics (purple lines). Although primarily discussed separately, many age-related chromatin changes are expected to be interconnected, as represented by the central thick arrows interconnecting all four main topics. Additional smaller arrows represent connections between subtopics as discussed in this review. Specifically, the CHD family of chromatin remodelers leads to changes in histone acetylation due to the inclusion of a histone deacetylase within the nucleosome remodeling and deacetylase (NuRD) complex (Basta and Rauchman, 2015). Changes in core histone protein levels may lead to changes in chromatin structure including inappropriately accessible DNA such as occurs with loss of heterochromatin, as suggested by Pal and Tyler (2016). Loss of heterochromatin and nuclear morphology changes are tightly linked and often seen together, as the structure anchoring heterochromatin to the nuclear periphery breaks down during aging (Gruenbaum et al., 2002). Loss of heterochromatin is also tightly linked with the heterochromatin-marking histone modifications H3K27me3 and H3K9me3, which are altered in many aging models (See “Loss of Heterochromatin”, “H3K27me3”, and “H3K9me3” sections of the text).

The chromatin environment is influenced by complex interactions between DNA, histones, and the proteins that regulate them. Changes at chromatin occur during aging, and evidence from model organisms suggests that factors involved in regulating chromatin can also modulate aging (See Figure 3; Supplementary Table S1 for summary). However, many of these results from studies of mutants must be taken with caution, as many chromatin factors or histone modifying enzymes can have non-chromatin targets or can impact multiple histone marks (Benayoun et al., 2015), and most studies do not demonstrate that the lifespan phenotype of a mutant is due to one particular action of that protein at chromatin. Furthermore, changes observed at chromatin during aging are not always consistent between different models and conditions, nor are perturbations that alter lifespan, as we have discussed examples of mutants that are long-lived under certain conditions, and short-lived under others. As chromatin is well-known to interact with the environment (Benayoun et al., 2015), this may not be completely surprising, but it does underlie the necessity to use extreme caution when extrapolating lifespan results beyond one particular condition, especially when considering the effects likely to be seen in different organisms. This will be especially important when considering any chromatin-targeting therapeutics to promote healthy aging or prevent disease in humans, as one treatment could have the potential to be either beneficial or detrimental depending on genetic background or environmental conditions.

As the field moves forward, it will be important for researchers to utilize careful analysis to further dissect the precise mechanisms that lead to lifespan modulation in different conditions. For example, many chromatin factors play important roles in development as well as longevity. Using perpetual mutants with developmental phenotypes or RNAi during development can confuse results and result in pleiotropic phenotypes. Additionally, the immediate consequence of factor loss at chromatin may be difficult to uncover, as many changes at chromatin and in gene expression may be secondary effects. Thus, carefully timed RNAi knockdown in adulthood or rapidly degradable protein tags such as the auxin-degradable system (Divekar et al., 2021) will be of great use to uncover the direct effects of chromatin factors and how and when they influence longevity.

Furthermore, the tissue of action for many chromatin factors remains unknown, and the effects of factor loss at a tissue-specific chromatin level is usually not studied. This remains an important area of study, as some factors are ubiquitously expressed but could play different roles at chromatin in different tissues, and understanding within which tissue they act to modulate lifespan could help to promote healthy aging while reducing negative side effects. Tissue specific knock-down and knock-in experiments are an important way to ask these questions, and as genomic techniques progress, assaying the chromatin environment in a tissue-specific manner, or ideally using single-cell approaches will provide the best insight as to precise changes occurring at chromatin in each cell. Combining cell-type specific changes in chromatin with single cell RNA-seq data will provide an excellent opportunity for precision. With the release of a single cell RNA-seq atlas of aging in *C. elegans* (Gao et al., 2023), the adaptation of single cell ATAC-seq to worms (Durham et al., 2021), and the development of single cell chromatin profiling tools (Bartosovic et al., 2021; Janssens et al., 2022), this type of resolution is becoming more feasible in *C. elegans*.

The aging biology field has made rapid progress over the last 30 years to uncover numerous mechanisms that modulate lifespan in diverse organisms. There is a clear link between chromatin and longevity, but much work remains to be done. As chromatin state is frequently considered to be plastic and modifications at chromatin as reversible, understanding the precise interplay between chromatin and longevity holds promise as a way to promote healthy aging.

## References

[B1] Aitlhadj L. Stürzenbaum S. R. (2010). The use of FUdR can cause prolonged longevity in mutant nematodes. Mech. Ageing Dev. 131, 364–365. 10.1016/j.mad.2010.03.002 20236608

[B2] Alvares S. M. Mayberry G. A. Joyner E. Y. Lakowski B. Ahmed S. (2014). H3K4 demethylase activities repress proliferative and postmitotic aging. Aging Cell 13, 245–253. 10.1111/acel.12166 24134677 PMC4020274

[B3] Andersen E. C. Lu X. Horvitz H. R. (2006). *C. elegans* ISWI and NURF301 antagonize an Rb-like pathway in the determination of multiple cell fates. Development 133, 2695–2704. 10.1242/dev.02444 16774993

[B4] Bannister A. J. Kouzarides T. (2011). Regulation of chromatin by histone modifications. Cell Res. 21, 381–395. 10.1038/cr.2011.22 21321607 PMC3193420

[B5] Bansal A. Kwon E.-S. Conte D. Jr Liu H. Gilchrist M. J. MacNeil L. T. (2014). Transcriptional regulation of *Caenorhabditis elegans* FOXO/DAF-16 modulates lifespan. Longev. Heal. 3, 5. 10.1186/2046-2395-3-5 PMC4022319 24834345

[B6] Bar D. Z. Gruenbaum Y. (2010). Reversal of age-dependent nuclear morphology by inhibition of prenylation does not affect lifespan in *Caenorhabditis elegans* . Nucleus 1, 499–505. 10.4161/nucl.1.6.13223 21327093 PMC3027053

[B7] Bar D. Z. Neufeld E. Feinstein N. Gruenbaum Y. (2009). Gliotoxin reverses age-dependent nuclear morphology phenotypes, ameliorates motility, but fails to affect lifespan of adult *Caenorhabditis elegans* . Cell Motil. Cytoskelet. 66, 791–797. 10.1002/cm.20347 19235201

[B8] Barnes V. L. Bhat A. Unnikrishnan A. Heydari A. R. Arking R. Pile L. A. (2014). SIN3 is critical for stress resistance and modulates adult lifespan. Aging 6, 645–660. 10.18632/aging.100684 25133314 PMC4169859

[B9] Bartosovic M. Kabbe M. Castelo-Branco G. (2021). Single-cell CUT&Tag profiles histone modifications and transcription factors in complex tissues. Nat. Biotechnol. 39, 825–835. 10.1038/s41587-021-00869-9 33846645 PMC7611252

[B10] Basta J. Rauchman M. (2015). The nucleosome remodeling and deacetylase complex in development and disease. Transl. Res. 165, 36–47. 10.1016/j.trsl.2014.05.003 24880148 PMC4793962

[B11] Bell C. G. Lowe R. Adams P. D. Baccarelli A. A. Beck S. Bell J. T. (2019). DNA methylation aging clocks: challenges and recommendations. Genome Biol. 20, 249. 10.1186/s13059-019-1824-y 31767039 PMC6876109

[B12] Benayoun B. A. Pollina E. A. Brunet A. (2015). Epigenetic regulation of ageing: linking environmental inputs to genomic stability. Nat. Rev. Mol. Cell Biol. 16, 593–610. 10.1038/nrm4048 26373265 PMC4736728

[B13] Berdichevsky A. Viswanathan M. Horvitz H. R. Guarente L. (2006). *C. elegans* SIR-2.1 interacts with 14-3-3 proteins to activate DAF-16 and extend life span. Cell 125, 1165–1177. 10.1016/j.cell.2006.04.036 16777605

[B14] Biswas S. Rao C. M. (2018). Epigenetic tools (The Writers, the Readers and the Erasers) and their implications in cancer therapy. Eur. J. Pharmacol. 837, 8–24. 10.1016/j.ejphar.2018.08.021 30125562

[B15] Booth L. N. Brunet A. (2016). The aging epigenome. Mol. Cell 62, 728–744. 10.1016/j.molcel.2016.05.013 27259204 PMC4917370

[B16] Bracken A. P. Kleine-Kohlbrecher D. Dietrich N. Pasini D. Gargiulo G. Beekman C. (2007). The Polycomb group proteins bind throughout the INK4A-ARF locus and are disassociated in senescent cells. Genes Dev. 21, 525–530. 10.1101/gad.415507 17344414 PMC1820894

[B17] Buchwalter A. Kaneshiro J. M. Hetzer M. W. (2019). Coaching from the sidelines: the nuclear periphery in genome regulation. Nat. Rev. Genet. 20, 39–50. 10.1038/s41576-018-0063-5 30356165 PMC6355253

[B18] Burnett C. Valentini S. Cabreiro F. Goss M. Somogyvári M. Piper M. D. (2011). Absence of effects of Sir2 overexpression on lifespan in *C. elegans* and Drosophila. Nature 477, 482–485. 10.1038/nature10296 21938067 PMC3188402

[B19] Capell B. C. Collins F. S. (2006). Human laminopathies: nuclei gone genetically awry. Nat. Rev. Genet. 7, 940–952. 10.1038/nrg1906 17139325

[B20] Caron M. Gely L. Garvis S. Adrait A. Couté Y. Palladino F. (2022). Loss of SET1/COMPASS methyltransferase activity reduces lifespan and fertility in *Caenorhabditis elegans* . Life Sci. Alliance 5. 10.26508/lsa.202101140 PMC8675910 34893559

[B21] Chandra T. Ewels P. A. Schoenfelder S. Furlan-Magaril M. Wingett S. W. Kirschner K. (2015). Global reorganization of the nuclear landscape in senescent cells. Cell Rep. 10, 471–483. 10.1016/j.celrep.2014.12.055 25640177 PMC4542308

[B22] Chandra T. Kirschner K. Thuret J.-Y. Pope B. D. Ryba T. Newman S. (2012). Independence of repressive histone marks and chromatin compaction during senescent heterochromatic layer formation. Mol. Cell 47, 203–214. 10.1016/j.molcel.2012.06.010 22795131 PMC3701408

[B23] Charar C. Metsuyanim-Cohen S. Bar D. Z. (2021). Lamin regulates the dietary restriction response via the mTOR pathway in *Caenorhabditis elegans* . J. Cell Sci. 134. 10.1242/jcs.258428 PMC8445603 34383046

[B24] Chen P. Li W. Li G. (2021). Structures and functions of chromatin fibers. Annu. Rev. Biophys. 50, 95–116. 10.1146/annurev-biophys-062920-063639 33957053

[B25] Chen Y. Bravo J. I. Son J. M. Lee C. Benayoun B. A. (2020). Remodeling of the H3 nucleosomal landscape during mouse aging. Transl. Med. Aging 4, 22–31. 10.1016/j.tma.2019.12.003 32462102 PMC7252472

[B26] Cheung P. Vallania F. Warsinske H. C. Donato M. Schaffert S. Chang S. E. (2018). Single-cell chromatin modification profiling reveals increased epigenetic variations with aging. Cell 173, 1385–1397.e14. 10.1016/j.cell.2018.03.079 29706550 PMC5984186

[B27] Clapier C. R. Cairns B. R. (2009). The biology of chromatin remodeling complexes. Annu. Rev. Biochem. 78, 273–304. 10.1146/annurev.biochem.77.062706.153223 19355820

[B28] Corpet A. Stucki M. (2014). Chromatin maintenance and dynamics in senescence: a spotlight on SAHF formation and the epigenome of senescent cells. Chromosoma 123, 423–436. 10.1007/s00412-014-0469-6 24861957

[B29] Crane M. M. Russell A. E. Schafer B. J. Blue B. W. Whalen R. Almazan J. (2019). DNA damage checkpoint activation impairs chromatin homeostasis and promotes mitotic catastrophe during aging. Elife 8. 10.7554/eLife.50778 PMC6850777 31714209

[B30] Cruz C. Della Rosa M. Krueger C. Gao Q. Horkai D. King M. (2018). Tri-methylation of histone H3 lysine 4 facilitates gene expression in ageing cells. Elife 7. 10.7554/eLife.34081 PMC6168286 30274593

[B31] Cui M. Han M. (2007). Roles of chromatin factors in *C. elegans* development. WormBook 2007, 1. 10.1895/wormbook.1.139.1 PMC4781364 18050494

[B32] Curran S. P. Wu X. Riedel C. G. Ruvkun G. (2009). A soma-to-germline transformation in long-lived *Caenorhabditis elegans* mutants. Nature 459, 1079–1084. 10.1038/nature08106 19506556 PMC2716045

[B33] Dang W. Steffen K. K. Perry R. Dorsey J. A. Johnson F. B. Shilatifard A. (2009). Histone H4 lysine 16 acetylation regulates cellular lifespan. Nature 459, 802–807. 10.1038/nature08085 19516333 PMC2702157

[B34] De Sandre-Giovannoli A. Bernard R. Cau P. Navarro C. Amiel J. Boccaccio I. (2003). Lamin a truncation in Hutchinson-Gilford progeria. Science 300, 2055. 10.1126/science.1084125 12702809

[B35] De Vaux V. Pfefferli C. Passannante M. Belhaj K. von Essen A. Sprecher S. G. (2013). The *Caenorhabditis elegans* LET-418/Mi2 plays a conserved role in lifespan regulation. Aging Cell 12, 1012–1020. 10.1111/acel.12129 23815345

[B36] Denzel M. S. Lapierre L. R. Mack H. I. D. (2019). Emerging topics in *C. elegans* aging research: transcriptional regulation, stress response and epigenetics. Mech. Ageing Dev. 177, 4–21. 10.1016/j.mad.2018.08.001 30134144 PMC6696993

[B37] Dittmer T. A. Misteli T. (2011). The lamin protein family. Genome Biol. 12, 222. 10.1186/gb-2011-12-5-222 21639948 PMC3219962

[B38] Divekar N. S. Horton H. E. Wignall S. M. (2021). Methods for rapid protein depletion in *C. elegans* using auxin-inducible degradation. Curr. Protoc. 1, e16. 10.1002/cpz1.16 33523606 PMC8767568

[B39] Djeghloul D. Kuranda K. Kuzniak I. Barbieri D. Naguibneva I. Choisy C. (2016). Age-associated decrease of the histone methyltransferase SUV39H1 in HSC perturbs heterochromatin and B lymphoid differentiation. Stem Cell Rep. 6, 970–984. 10.1016/j.stemcr.2016.05.007 PMC4911502 27304919

[B40] Durham T. J. Daza R. M. Gevirtzman L. Cusanovich D. A. Bolonduro O. Noble W. S. (2021). Comprehensive characterization of tissue-specific chromatin accessibility in L2 *Caenorhabditis elegans* nematodes. Genome Res. 31, 1952–1969. 10.1101/gr.271791.120 33888511 PMC8494234

[B41] Edwards C. Canfield J. Copes N. Rehan M. Lipps D. Bradshaw P. C. (2014). D-beta-hydroxybutyrate extends lifespan in *C. elegans* . Aging 6, 621–644. 10.18632/aging.100683 25127866 PMC4169858

[B42] Eissenberg J. C. Shilatifard A. (2006). Leaving a mark: the many footprints of the elongating RNA polymerase II. Curr. Opin. Genet. Dev. 16, 184–190. 10.1016/j.gde.2006.02.004 16503129

[B43] Emerson F. J. Chiu C. Lin L. Y. Riedel C. G. Zhu M. Lee S. S. (2023). The chromatin factors SET-26 and HCF-1 oppose the histone deacetylase HDA-1 in longevity and gene regulation in *C. elegans* . bioRxiv. 10.1101/2023.03.20.531974 PMC10940595 38485937

[B44] Emerson F. J. (2023). Dissecting the molecular mechanisms of longevity-regulating chromatin factors in *C. elegans*. Dissertation. Ithaca (NY): Cornell University.

[B45] Eriksson M. Brown W. T. Gordon L. B. Glynn M. W. Singer J. Scott L. (2003). Recurrent de novo point mutations in lamin A cause Hutchinson–Gilford progeria syndrome. Nature 423, 293–298. 10.1038/nature01629 12714972 PMC10540076

[B46] Fan Q. Li X.-M. Zhai C. Li B. Li S.-T. Dong M.-Q. (2023). Somatic nuclear blebbing in *Caenorhabditis elegans* is not a feature of organismal aging but a potential indicator of germline proliferation in early adulthood. G3 (Bethesda) 13, jkad029. 10.1093/g3journal/jkad029 36735812 PMC10085788

[B47] Feser J. Truong D. Das C. Carson J. J. Kieft J. Harkness T. (2010). Elevated histone expression promotes life span extension. Mol. Cell 39, 724–735. 10.1016/j.molcel.2010.08.015 20832724 PMC3966075

[B48] Fong N. Saldi T. Sheridan R. M. Cortazar M. A. Bentley D. L. (2017). RNA pol II dynamics modulate Co-transcriptional chromatin modification, CTD phosphorylation, and transcriptional direction. Mol. Cell 66, 546–557.e3. 10.1016/j.molcel.2017.04.016 28506463 PMC5488731

[B49] Gao S. M. Qi Y. Zhang Q. Mohammed A. S. Lee Y.-T. Guan Y. (2023). Aging atlas reveals cell-type-specific regulation of pro-longevity strategies. bioRxiv. 10.1101/2023.02.28.530490 PMC11257944 38816550

[B50] Garay E. Campos S. E. González de la Cruz J. Gaspar A. P. Jinich A. Deluna A. (2014). High-resolution profiling of stationary-phase survival reveals yeast longevity factors and their genetic interactions. PLoS Genet. 10, e1004168. 10.1371/journal.pgen.1004168 24586198 PMC3937222

[B51] Golden N. L. Foley M. K. Kim Guisbert K. S. Guisbert E. (2022). Divergent regulatory roles of NuRD chromatin remodeling complex subunits GATAD2 and CHD4 in *Caenorhabditis elegans* . Genetics 221. 10.1093/genetics/iyac046 PMC9071545 35323946

[B52] Goldman R. D. Shumaker D. K. Erdos M. R. Eriksson M. Goldman A. E. Gordon L. B. (2004). Accumulation of mutant lamin A causes progressive changes in nuclear architecture in Hutchinson–Gilford progeria syndrome. Proc. Natl. Acad. Sci. 101, 8963–8968. 10.1073/pnas.0402943101 15184648 PMC428455

[B53] Greer E. L. Becker B. Latza C. Antebi A. Shi Y. (2016). Mutation of *C. elegans* demethylase spr-5 extends transgenerational longevity. Cell Res. 26, 229–238. 10.1038/cr.2015.148 26691751 PMC4746603

[B54] Greer E. L. Blanco M. A. Gu L. Sendinc E. Liu J. Aristizábal-Corrales D. (2015). DNA methylation on N6-adenine in *C. elegans* . Cell 161, 868–878. 10.1016/j.cell.2015.04.005 25936839 PMC4427530

[B55] Greer E. L. Maures T. J. Hauswirth A. G. Green E. M. Leeman D. S. Maro G. S. (2010). Members of the H3K4 trimethylation complex regulate lifespan in a germline-dependent manner in *C. elegans* . Nature 466, 383–387. 10.1038/nature09195 20555324 PMC3075006

[B56] Greer E. L. Maures T. J. Ucar D. Hauswirth A. G. Mancini E. Lim J. P. (2011). Transgenerational epigenetic inheritance of longevity in *Caenorhabditis elegans* . Nature 479, 365–371. 10.1038/nature10572 22012258 PMC3368121

[B57] Gruenbaum Y. Moir R. D. Shumaker D. K. (2002). Nuclear lamins: building blocks of nuclear architecture. Genes Dev. 16, 533. 10.1101/gad.960502 11877373

[B58] Guillermo A. R. R. Chocian K. Gavriilidis G. Vandamme J. Salcini A. E. Mellor J. (2021). H3K27 modifiers regulate lifespan in *C. elegans* in a context-dependent manner. BMC Biol. 19, 59. 10.1186/s12915-021-00984-8 33766022 PMC7995591

[B59] Haithcock E. Dayani Y. Neufeld E. Zahand A. J. Feinstein N. Mattout A. (2005). Age-related changes of nuclear architecture in *Caenorhabditis elegans* . Proc. Natl. Acad. Sci. U. S. A. 102, 16690–16695. 10.1073/pnas.0506955102 16269543 PMC1283819

[B60] Hamilton B. Dong Y. Shindo M. Liu W. Odell I. Ruvkun G. (2005). A systematic RNAi screen for longevity genes in *C. elegans* . Genes Dev. 19, 1544–1555. 10.1101/gad.1308205 15998808 PMC1172061

[B61] Han S. Schroeder E. A. Silva-García C. G. Hebestreit K. Mair W. B. Brunet A. (2017). Mono-unsaturated fatty acids link H3K4me3 modifiers to *C. elegans* lifespan. Nature 544, 185–190. 10.1038/nature21686 28379943 PMC5391274

[B62] Henikoff S. (2008). Nucleosome destabilization in the epigenetic regulation of gene expression. Nat. Rev. Genet. 9, 15–26. 10.1038/nrg2206 18059368

[B63] Herranz D. Muñoz-Martin M. Cañamero M. Mulero F. Martinez-Pastor B. Fernandez-Capetillo O. (2010). Sirt1 improves healthy ageing and protects from metabolic syndrome-associated cancer. Nat. Commun. 1, 3. 10.1038/ncomms1001 20975665 PMC3641391

[B64] Herranz D. Serrano M. (2010). SIRT1: recent lessons from mouse models. Nat. Rev. Cancer 10, 819–823. 10.1038/nrc2962 21102633 PMC3672967

[B65] Horvath S. (2013). DNA methylation age of human tissues and cell types. Genome Biol. 14, R115. 10.1186/gb-2013-14-10-r115 24138928 PMC4015143

[B66] Horvath S. (2015). Erratum to: DNA methylation age of human tissues and cell types. Genome Biol. 16, 96. 10.1186/s13059-015-0649-6 25968125 PMC4427927

[B67] Hu Z. Chen K. Xia Z. Chavez M. Pal S. Seol J.-H. (2014). Nucleosome loss leads to global transcriptional up-regulation and genomic instability during yeast aging. Genes Dev. 28, 396–408. 10.1101/gad.233221.113 24532716 PMC3937517

[B68] Huang M. Hong M. Hou X. Zhu C. Chen D. Chen X. (2022). H3K9me1/2 methylation limits the lifespan of daf-2 mutants in *C. elegans* . Elife 11. 10.7554/eLife.74812 PMC9514849 36125117

[B69] Ito T. Teo Y. V. Evans S. A. Neretti N. Sedivy J. M. (2018). Regulation of cellular senescence by Polycomb chromatin modifiers through distinct DNA damage- and histone methylation-dependent pathways. Cell Rep. 22, 3480–3492. 10.1016/j.celrep.2018.03.002 29590617 PMC5915310

[B70] Ivanov A. Pawlikowski J. Manoharan I. van Tuyn J. Nelson D. M. Rai T. S. (2013). Lysosome-mediated processing of chromatin in senescence. J. Cell Biol. 202, 129–143. 10.1083/jcb.201212110 23816621 PMC3704985

[B71] Janssens D. H. Otto D. J. Meers M. P. Setty M. Ahmad K. Henikoff S. (2022). CUT&Tag2for1: a modified method for simultaneous profiling of the accessible and silenced regulome in single cells. Genome Biol. 23, 81. 10.1186/s13059-022-02642-w 35300717 PMC8928696

[B72] Jin C. Li J. Green C. D. Yu X. Tang X. Han D. (2011). Histone demethylase UTX-1 regulates *C. elegans* life span by targeting the insulin/IGF-1 signaling pathway. Cell Metab. 14, 161–172. 10.1016/j.cmet.2011.07.001 21803287

[B73] Jofré D. M. Hoffman D. K. Cervino A. S. Hahn G. M. Grundy M. Yun S. (2022). The CHARGE syndrome ortholog CHD-7 regulates TGF-β pathways in *Caenorhabditis elegans* . Proc. Natl. Acad. Sci. U. S. A. 119, e2109508119. 10.1073/pnas.2109508119 35394881 PMC9169646

[B74] Johnson D. S. Mortazavi A. Myers R. M. Wold B. (2007). Genome-wide mapping of *in vivo* protein-DNA interactions. Science 316, 1497–1502. 10.1126/science.1141319 17540862

[B75] Jung M. Pfeifer G. P. (2015). Aging and DNA methylation. BMC Biol. 13, 7. 10.1186/s12915-015-0118-4 25637097 PMC4311512

[B76] Kaeberlein M. McVey M. Guarente L. (1999). The SIR2/3/4 complex and SIR2 alone promote longevity in *Saccharomyces cerevisiae* by two different mechanisms. Genes Dev. 13, 2570–2580. 10.1101/gad.13.19.2570 10521401 PMC317077

[B77] Kawamura K. Maruyama I. N. (2020). Mutation in histone deacetylase HDA-3 leads to shortened locomotor healthspan in *Caenorhabditis elegans* . Aging 12, 23525–23547. 10.18632/aging.202296 33276344 PMC7762513

[B78] Kemphues K. J. Kusch M. Wolf N. (1988). Maternal-effect lethal mutations on linkage group II of *Caenorhabditis elegans* . Genetics 120, 977–986. 10.1093/genetics/120.4.977 3224814 PMC1203589

[B79] Kenyon C. Chang J. Gensch E. Rudner A. Tabtiang R. (1993). A *C. elegans* mutant that lives twice as long as wild type. Nature 366, 461–464. 10.1038/366461a0 8247153

[B80] Klar A. J. Fogel S. (1979). Activation of mating type genes by transposition in *Saccharomyces cerevisiae* . Proc. Natl. Acad. Sci. U. S. A. 76, 4539–4543. 10.1073/pnas.76.9.4539 388445 PMC411613

[B81] Kouzarides T. (2007). Chromatin modifications and their function. Cell 128, 693–705. 10.1016/j.cell.2007.02.005 17320507

[B82] Kuzmanov A. Karina E. I. Kirienko N. V. Fay D. S. (2014). The conserved PBAF nucleosome-remodeling complex mediates the response to stress in *Caenorhabditis elegans* . Mol. Cell. Biol. 34, 1121–1135. 10.1128/MCB.01502-13 24421384 PMC3958046

[B83] Labbadia J. Morimoto R. I. (2015). Repression of the heat shock response is a programmed event at the onset of reproduction. Mol. Cell 59, 639–650. 10.1016/j.molcel.2015.06.027 26212459 PMC4546525

[B84] Lapasset L. Milhavet O. Prieur A. Besnard E. Babled A. Aït-Hamou N. (2011). Rejuvenating senescent and centenarian human cells by reprogramming through the pluripotent state. Genes & Dev. 25, 2248–2253. 10.1101/gad.173922.111 22056670 PMC3219229

[B85] Large E. E. Xu W. Zhao Y. Brady S. C. Long L. Butcher R. A. (2016). Selection on a subunit of the NURF chromatin remodeler modifies life history traits in a domesticated strain of *Caenorhabditis elegans* . PLoS Genet. 12, e1006219. 10.1371/journal.pgen.1006219 27467070 PMC4965130

[B86] Larson K. Yan S.-J. Tsurumi A. Liu J. Zhou J. Gaur K. (2012). Heterochromatin formation promotes longevity and represses ribosomal RNA synthesis. PLoS Genet. 8, e1002473. 10.1371/journal.pgen.1002473 22291607 PMC3266895

[B87] Lee S. S. Kennedy S. Tolonen A. C. Ruvkun G. (2003). DAF-16 target genes that control *C. elegans* life-span and metabolism. Science 300, 644–647. 10.1126/science.1083614 12690206

[B88] Lee T. W.-S. David H. S. Engstrom A. K. Carpenter B. S. Katz D. J. (2019). Repressive H3K9me2 protects lifespan against the transgenerational burden of COMPASS activity in *C. elegans* . Elife 8. 10.7554/eLife.48498 PMC7299346 31815663

[B89] Li C.-L. Pu M. Wang W. Chaturbedi A. Emerson F. J. Lee S. S. (2021). Region-specific H3K9me3 gain in aged somatic tissues in *Caenorhabditis elegans* . PLoS Genet. 17, e1009432. 10.1371/journal.pgen.1009432 34506495 PMC8457455

[B90] Li J. Ebata A. Dong Y. Rizki G. Iwata T. Lee S. S. (2008). *Caenorhabditis elegans* HCF-1 functions in longevity maintenance as a DAF-16 regulator. PLoS Biol. 6, e233. 10.1371/journal.pbio.0060233 18828672 PMC2553839

[B91] Li L. Greer C. Eisenman R. N. Secombe J. (2010). Essential functions of the histone demethylase lid. PLoS Genet. 6, e1001221. 10.1371/journal.pgen.1001221 21124823 PMC2991268

[B92] Lieberman-Aiden E. van Berkum N. L. Williams L. Imakaev M. Ragoczy T. Telling A. (2009). Comprehensive mapping of long-range interactions reveals folding principles of the human genome. Science 326, 289–293. 10.1126/science.1181369 19815776 PMC2858594

[B93] Liu B. Wang Z. Zhang L. Ghosh S. Zheng H. Zhou Z. (2013a). Depleting the methyltransferase Suv39h1 improves DNA repair and extends lifespan in a progeria mouse model. Nat. Commun. 4, 1868. 10.1038/ncomms2885 23695662 PMC3674265

[B94] Liu L. Cheung T. H. Charville G. W. Hurgo B. M. C. Leavitt T. Shih J. (2013b). Chromatin modifications as determinants of muscle stem cell quiescence and chronological aging. Cell Rep. 4, 189–204. 10.1016/j.celrep.2013.05.043 23810552 PMC4103025

[B95] López-Otín C. Blasco M. A. Partridge L. Serrano M. Kroemer G. (2013). The hallmarks of aging. Cell 153, 1194–1217. 10.1016/j.cell.2013.05.039 23746838 PMC3836174

[B96] Mannervik M. Levine M. (1999). The Rpd3 histone deacetylase is required for segmentation of the Drosophila embryo. Proc. Natl. Acad. Sci. U. S. A. 96, 6797–6801. 10.1073/pnas.96.12.6797 10359792 PMC21995

[B97] Matilainen O. Sleiman M. S. B. Quiros P. M. Garcia S. M. D. A. Auwerx J. (2017). The chromatin remodeling factor ISW-1 integrates organismal responses against nuclear and mitochondrial stress. Nat. Commun. 8, 1818. 10.1038/s41467-017-01903-8 29180639 PMC5703887

[B98] Maures T. J. Greer E. L. Hauswirth A. G. Brunet A. (2011). The H3K27 demethylase UTX-1 regulates *C. elegans* lifespan in a germline-independent, insulin-dependent manner. Aging Cell 10, 980–990. 10.1111/j.1474-9726.2011.00738.x 21834846 PMC3215905

[B99] Maze I. Wenderski W. Noh K.-M. Bagot R. C. Tzavaras N. Purushothaman I. (2015). Critical role of histone turnover in neuronal transcription and plasticity. Neuron 87, 77–94. 10.1016/j.neuron.2015.06.014 26139371 PMC4491146

[B100] McCauley B. S. Sun L. Yu R. Lee M. Liu H. Leeman D. S. (2021). Altered chromatin states drive cryptic transcription in aging mammalian stem cells. Nat. Aging 1, 684–697. 10.1038/s43587-021-00091-x 34746802 PMC8570536

[B101] Meister P. Schott S. Bedet C. Xiao Y. Rohner S. Bodennec S. (2011). *Caenorhabditis elegans* Heterochromatin protein 1 (HPL-2) links developmental plasticity, longevity and lipid metabolism. Genome Biol. 12, R123. 10.1186/gb-2011-12-12-r123 22185090 PMC3334618

[B102] Merkwirth C. Jovaisaite V. Durieux J. Matilainen O. Jordan S. D. Quiros P. M. (2016). Two conserved histone demethylases regulate mitochondrial stress-induced longevity. Cell 165, 1209–1223. 10.1016/j.cell.2016.04.012 27133168 PMC4889222

[B103] Mertens J. Paquola A. C. M. Ku M. Hatch E. Böhnke L. Ladjevardi S. (2015). Directly reprogrammed human neurons retain aging-associated transcriptomic signatures and reveal age-related nucleocytoplasmic defects. Cell Stem Cell 17, 705–718. 10.1016/j.stem.2015.09.001 26456686 PMC5929130

[B104] Milazzo G. Mercatelli D. Di Muzio G. Triboli L. De Rosa P. Perini G. (2020). Histone deacetylases (HDACs): evolution, specificity, role in transcriptional complexes, and pharmacological actionability. Genes 11. 10.3390/genes11050556 PMC7288346 32429325

[B105] Min J.-N. Tian Y. Xiao Y. Wu L. Li L. Chang S. (2013). The mINO80 chromatin remodeling complex is required for efficient telomere replication and maintenance of genome stability. Cell Res. 23, 1396–1413. 10.1038/cr.2013.113 23979016 PMC3847565

[B106] Morgan M. A. J. Shilatifard A. (2020). Reevaluating the roles of histone-modifying enzymes and their associated chromatin modifications in transcriptional regulation. Nat. Genet. 52, 1271–1281. 10.1038/s41588-020-00736-4 33257899

[B107] Mosevitsky M. I. (2022). Progerin and its role in accelerated and natural aging. Mol. Biol. 56, 125–146. 10.1134/S0026893322020091 35403615

[B108] Murphy C. T. (2006). The search for DAF-16/FOXO transcriptional targets: approaches and discoveries. Exp. Gerontol. 41, 910–921. 10.1016/j.exger.2006.06.040 16934425

[B109] Müthel S. Uyar B. He M. Krause A. Vitrinel B. Bulut S. (2019). The conserved histone chaperone LIN-53 is required for normal lifespan and maintenance of muscle integrity in *Caenorhabditis elegans* . Aging Cell 18, e13012. 10.1111/acel.13012 31397537 PMC6826145

[B110] Narayan V. Ly T. Pourkarimi E. Murillo A. B. Gartner A. Lamond A. I. (2016). Deep proteome analysis identifies age-related processes in *C. elegans* . Cell Syst. 3, 144–159. 10.1016/j.cels.2016.06.011 27453442 PMC5003814

[B111] Narita M. Nũnez S. Heard E. Narita M. Lin A. W. Hearn S. A. (2003). Rb-mediated heterochromatin formation and silencing of E2F target genes during cellular senescence. Cell 113, 703–716. 10.1016/s0092-8674(03)00401-x 12809602

[B112] Ni Z. Ebata A. Alipanahiramandi E. Lee S. S. (2012). Two SET domain containing genes link epigenetic changes and aging in *Caenorhabditis elegans* . Aging Cell 11, 315–325. 10.1111/j.1474-9726.2011.00785.x 22212395 PMC3306474

[B113] Nikooei T. McDonagh A. M van der Linden A. (2020). The salt-inducible kinase KIN-29 regulates lifespan via the class II histone-deacetylase HDA-4. Micropubl. Biol. 2020. 10.17912/micropub.biology.000289 PMC7581299 33111058

[B114] Oberdoerffer P. Michan S. McVay M. Mostoslavsky R. Vann J. Park S.-K. (2008). SIRT1 redistribution on chromatin promotes genomic stability but alters gene expression during aging. Cell 135, 907–918. 10.1016/j.cell.2008.10.025 19041753 PMC2853975

[B115] O’Sullivan R. J. Kubicek S. Schreiber S. L. Karlseder J. (2010). Reduced histone biosynthesis and chromatin changes arising from a damage signal at telomeres. Nat. Struct. Mol. Biol. 17, 1218–1225. 10.1038/nsmb.1897 20890289 PMC2951278

[B116] Pal S. Tyler J. K. (2016). Epigenetics and aging. Sci. Adv. 2, e1600584. 10.1126/sciadv.1600584 27482540 PMC4966880

[B117] Pandey R. Sharma M. Saluja D. (2018). SIN-3 as a key determinant of lifespan and its sex dependent differential role on healthspan in *Caenorhabditis elegans* . Aging 10, 3910–3937. 10.18632/aging.101682 30541942 PMC6326684

[B118] Park J. Belden W. J. (2018). Long non-coding RNAs have age-dependent diurnal expression that coincides with age-related changes in genome-wide facultative heterochromatin. BMC Genomics 19, 777. 10.1186/s12864-018-5170-3 30373515 PMC6206985

[B119] Park S.-Y. Kim J.-S. (2020). A short guide to histone deacetylases including recent progress on class II enzymes. Exp. Mol. Med. 52, 204–212. 10.1038/s12276-020-0382-4 32071378 PMC7062823

[B120] Passannante M. Marti C.-O. Pfefferli C. Moroni P. S. Kaeser-Pebernard S. Puoti A. (2010). Different Mi-2 complexes for various developmental functions in *Caenorhabditis elegans* . PLoS One 5, e13681. 10.1371/journal.pone.0013681 21060680 PMC2965115

[B121] Penagos-Puig A. Furlan-Magaril M. (2020). Heterochromatin as an important driver of genome organization. Front. Cell Dev. Biol. 8, 579137. 10.3389/fcell.2020.579137 33072761 PMC7530337

[B122] Pérez-Jiménez M. M. Rodríguez-Palero M. J. Ródenas E. Askjaer P. Muñoz M. J. (2014). Age-dependent changes of nuclear morphology are uncoupled from longevity in *Caenorhabditis elegans* IGF/insulin receptor daf-2 mutants. Biogerontology 15, 279–288. 10.1007/s10522-014-9497-0 24671263

[B123] Piazzesi A. Papić D. Bertan F. Salomoni P. Nicotera P. Bano D. (2016). Replication-independent histone variant H3.3 controls animal lifespan through the regulation of pro-longevity transcriptional programs. Cell Rep. 17, 987–996. 10.1016/j.celrep.2016.09.074 27760329 PMC5081402

[B124] Price A. J. Manjegowda M. C. Kain J. Anandh S. Bochkis I. M. (2020). Hdac3, Setdb1, and Kap1 mark H3K9me3/H3K14ac bivalent regions in young and aged liver. Aging Cell 19, e13092. 10.1111/acel.13092 31858687 PMC6996956

[B125] Prieur A. Besnard E. Babled A. Lemaitre J.-M. (2011). p53 and p16INK4A independent induction of senescence by chromatin-dependent alteration of S-phase progression. Nat. Commun. 2, 1–10. 10.1038/ncomms1473 21915115

[B126] Pu M. Ni Z. Wang M. Wang X. Wood J. G. Helfand S. L. (2015). Trimethylation of Lys36 on H3 restricts gene expression change during aging and impacts life span. Genes & Dev. 29, 718–731. 10.1101/gad.254144.114 25838541 PMC4387714

[B127] Pu M. Wang M. Wang W. Velayudhan S. S. Lee S. S. (2018). Unique patterns of trimethylation of histone H3 lysine 4 are prone to changes during aging in *Caenorhabditis elegans* somatic cells. PLoS Genet. 14, e1007466. 10.1371/journal.pgen.1007466 29912876 PMC6023244

[B128] Remolina S. C. Hughes K. A. (2008). Evolution and mechanisms of long life and high fertility in queen honey bees. Age 30, 177–185. 10.1007/s11357-008-9061-4 19424867 PMC2527632

[B129] Revtovich A. V. Lee R. Kirienko N. V. (2019). Interplay between mitochondria and diet mediates pathogen and stress resistance in *Caenorhabditis elegans* . PLOS Genet. 15, e1008011. 10.1371/journal.pgen.1008011 30865620 PMC6415812

[B130] Riedel C. G. Dowen R. H. Lourenco G. F. Kirienko N. V. Heimbucher T. West J. A. (2013). DAF-16 employs the chromatin remodeller SWI/SNF to promote stress resistance and longevity. Nat. Cell Biol. 15, 491–501. 10.1038/ncb2720 23604319 PMC3748955

[B131] Rizki G. Iwata T. N. Li J. Riedel C. G. Picard C. L. Jan M. (2011). The evolutionarily conserved longevity determinants HCF-1 and SIR-2.1/SIRT1 collaborate to regulate DAF-16/FOXO. PLoS Genet. 7, e1002235. 10.1371/journal.pgen.1002235 21909281 PMC3164695

[B132] Rogakou E. P. Sekeri-Pataryas K. E. (1999). Histone variants of H2A and H3 families are regulated during *in vitro* aging in the same manner as during differentiation. Exp. Gerontol. 34, 741–754. 10.1016/s0531-5565(99)00046-7 10579635

[B133] Rogina B. Helfand S. L. Frankel S. (2002). Longevity regulation by Drosophila Rpd3 deacetylase and caloric restriction. Science 298, 1745. 10.1126/science.1078986 12459580

[B134] Rogina B. Helfand S. L. (2004). Sir2 mediates longevity in the fly through a pathway related to calorie restriction. Proc. Natl. Acad. Sci. U. S. A. 101, 15998–16003. 10.1073/pnas.0404184101 15520384 PMC528752

[B135] Rooney J. P. Luz A. L. González-Hunt C. P. Bodhicharla R. Ryde I. T. Anbalagan C. (2014). Effects of 5′-fluoro-2-deoxyuridine on mitochondrial biology in *Caenorhabditis elegans* . Exp. Gerontol. 56, 69–76. 10.1016/j.exger.2014.03.021 24704715 PMC4048797

[B136] Satoh A. Brace C. S. Rensing N. Cliften P. Wozniak D. F. Herzog E. D. (2013). Sirt1 extends life span and delays aging in mice through the regulation of Nk2 homeobox 1 in the DMH and LH. Cell Metab. 18, 416–430. 10.1016/j.cmet.2013.07.013 24011076 PMC3794712

[B137] Saul D. Kosinsky R. L. (2021). Epigenetics of aging and aging-associated diseases. Int. J. Mol. Sci. 22. 10.3390/ijms22010401 PMC7794926 33401659

[B138] Saunders A. Core L. J. Lis J. T. (2006). Breaking barriers to transcription elongation. Nat. Rev. Mol. Cell Biol. 7, 557–567. 10.1038/nrm1981 16936696

[B139] Scaffidi P. Misteli T. (2006). Lamin A-dependent nuclear defects in human aging. Science 312, 1059–1063. 10.1126/science.1127168 16645051 PMC1855250

[B140] Sen P. Dang W. Donahue G. Dai J. Dorsey J. Cao X. (2015). H3K36 methylation promotes longevity by enhancing transcriptional fidelity. Genes Dev. 29, 1362–1376. 10.1101/gad.263707.115 26159996 PMC4511212

[B141] Shah P. P. Donahue G. Otte G. L. Capell B. C. Nelson D. M. Cao K. (2013). Lamin B1 depletion in senescent cells triggers large-scale changes in gene expression and the chromatin landscape. Genes Dev. 27, 1787–1799. 10.1101/gad.223834.113 23934658 PMC3759695

[B142] Shao L.-W. Peng Q. Dong M. Gao K. Li Y. Li Y. (2020). Histone deacetylase HDA-1 modulates mitochondrial stress response and longevity. Nat. Commun. 11, 4639. 10.1038/s41467-020-18501-w 32934238 PMC7493924

[B143] Sharma M. Pandey R. Saluja D. (2018). ROS is the major player in regulating altered autophagy and lifespan in sin-3 mutants of *C. elegans* . Autophagy 14, 1239–1255. 10.1080/15548627.2018.1474312 29912629 PMC6103711

[B144] Shumaker D. K. Dechat T. Kohlmaier A. Adam S. A. Bozovsky M. R. Erdos M. R. (2006). Mutant nuclear lamin A leads to progressive alterations of epigenetic control in premature aging. Proc. Natl. Acad. Sci. U. S. A. 103, 8703–8708. 10.1073/pnas.0602569103 16738054 PMC1472659

[B145] Siebold A. P. Banerjee R. Tie F. Kiss D. L. Moskowitz J. Harte P. J. (2010). Polycomb Repressive Complex 2 and Trithorax modulate Drosophila longevity and stress resistance. Proc. Natl. Acad. Sci. U. S. A. 107, 169–174. 10.1073/pnas.0907739107 20018689 PMC2806727

[B146] Silva-García C. G. Mair W. B. (2022). Confirming the pro-longevity effects of H3K4me3-deficient set-2 mutants in extending lifespan in *C. elegans* . bioRxiv. 10.1101/2022.08.02.502497

[B147] Smeal T. Claus J. Kennedy B. Cole F. Guarente L. (1996). Loss of transcriptional silencing causes sterility in old mother cells of *S. cerevisiae* . Cell 84, 633–642. 10.1016/s0092-8674(00)81038-7 8598049

[B148] Spracklin G. Fields B. Wan G. Becker D. Wallig A. Shukla A. (2017). The RNAi inheritance machinery of *Caenorhabditis elegans* . Genetics 206, 1403–1416. 10.1534/genetics.116.198812 28533440 PMC5500139

[B149] Stuhr N. L. Curran S. P. (2020). Bacterial diets differentially alter lifespan and healthspan trajectories in *C. elegans* . Commun. Biol. 3, 653. 10.1038/s42003-020-01379-1 33159120 PMC7648844

[B150] Sun D. Luo M. Jeong M. Rodriguez B. Xia Z. Hannah R. (2014). Epigenomic profiling of young and aged HSCs reveals concerted changes during aging that reinforce self-renewal. Cell Stem Cell 14, 673–688. 10.1016/j.stem.2014.03.002 24792119 PMC4070311

[B151] Sural S. Liang C.-Y. Wang F.-Y. Ching T.-T. Hsu A.-L. (2020). HSB-1/HSF-1 pathway modulates histone H4 in mitochondria to control mtDNA transcription and longevity. Sci. Adv. 6. 10.1126/sciadv.aaz4452 PMC7577724 33087356

[B152] Swer P. B. Sharma R. (2021). ATP-dependent chromatin remodelers in ageing and age-related disorders. Biogerontology 22, 1–17. 10.1007/s10522-020-09899-3 32968929

[B153] Tamaru H. (2010). Confining euchromatin/heterochromatin territory: jumonji crosses the line. Genes Dev. 24, 1465–1478. 10.1101/gad.1941010 20634313 PMC2904936

[B154] Tissenbaum H. A. Guarente L. (2001). Increased dosage of a sir-2 gene extends lifespan in *Caenorhabditis elegans* . Nature 410, 227–230. 10.1038/35065638 11242085

[B155] Tomimatsu K. Bihary D. Olan I. Parry A. J. Schoenfelder S. Chan A. S. L. (2021). Locus-specific induction of gene expression from heterochromatin loci during cellular senescence. Nat. Aging 2, 31–45. 10.1038/s43587-021-00147-y 37118356

[B156] Tonoyama Y. Shinya M. Toyoda A. Kitano T. Oga A. Nishimaki T. (2018). Abnormal nuclear morphology is independent of longevity in a zmpste24-deficient fish model of Hutchinson-Gilford progeria syndrome (HGPS). Comp. Biochem. Physiol. C. Toxicol. Pharmacol. 209, 54–62. 10.1016/j.cbpc.2018.03.006 29567411

[B157] Tsurumi A. Li W. X. (2012). Global heterochromatin loss: a unifying theory of aging? Epigenetics 7, 680–688. 10.4161/epi.20540 22647267 PMC3414389

[B158] Tvardovskiy A. Schwämmle V. Kempf S. J. Rogowska-Wrzesinska A. Jensen O. N. (2017). Accumulation of histone variant H3.3 with age is associated with profound changes in the histone methylation landscape. Nucleic Acids Res. 45, 9272–9289. 10.1093/nar/gkx696 28934504 PMC5766163

[B159] Unnikrishnan A. Freeman W. M. Jackson J. Wren J. D. Porter H. Richardson A. (2019). The role of DNA methylation in epigenetics of aging. Pharmacol. Ther. 195, 172–185. 10.1016/j.pharmthera.2018.11.001 30419258 PMC6397707

[B160] van der Linden A. M. Nolan K. M. Sengupta P. (2007). KIN-29 SIK regulates chemoreceptor gene expression via an MEF2 transcription factor and a class II HDAC. EMBO J. 26, 358–370. 10.1038/sj.emboj.7601479 17170704 PMC1783467

[B161] Van Raamsdonk J. M. Hekimi S. (2011). FUdR causes a twofold increase in the lifespan of the mitochondrial mutant gas-1. Mech. Ageing Dev. 132, 519–521. 10.1016/j.mad.2011.08.006 21893079 PMC4074524

[B162] Varela I. Pereira S. Ugalde A. P. Navarro C. L. Suárez M. F. Cau P. (2008). Combined treatment with statins and aminobisphosphonates extends longevity in a mouse model of human premature aging. Nat. Med. 14, 767–772. 10.1038/nm1786 18587406

[B163] Villeponteau B. (1997). The heterochromatin loss model of aging. Exp. Gerontol. 32, 383–394. 10.1016/s0531-5565(96)00155-6 9315443

[B164] Viswanathan M. Guarente L. (2011). Regulation of *Caenorhabditis elegans* lifespan by sir-2.1 transgenes. Nature 477, E1–E2. 10.1038/nature10440 21938026

[B165] Viswanathan M. Kim S. K. Berdichevsky A. Guarente L. (2005). A role for SIR-2.1 regulation of ER stress response genes in determining *C. elegans* life span. Dev. Cell 9, 605–615. 10.1016/j.devcel.2005.09.017 16256736

[B166] Wan Q.-L. Meng X. Dai W. Luo Z. Wang C. Fu X. (2021). N^6^-methyldeoxyadenine and histone methylation mediate transgenerational survival advantages induced by hormetic heat stress. Sci. Adv. 7. 10.1126/sciadv.abc3026 PMC7775758 33523838

[B167] Wan Q.-L. Meng X. Wang C. Dai W. Luo Z. Yin Z. (2022). Histone H3K4me3 modification is a transgenerational epigenetic signal for lipid metabolism in *Caenorhabditis elegans* . Nat. Commun. 13, 768. 10.1038/s41467-022-28469-4 35140229 PMC8828817

[B168] Wang H. Liu Z. Wang Y. Ma L. Zhang W. Xu B. (2020). Genome-wide differential DNA methylation in reproductive, morphological, and visual system differences between queen bee and worker bee (*Apis mellifera*). Front. Genet. 11, 770. 10.3389/fgene.2020.00770 32903639 PMC7438783

[B169] Wang H. Zhao Y. Zhang Z. (2019). Age-dependent effects of floxuridine (FUdR) on senescent pathology and mortality in the nematode *Caenorhabditis elegans* . Biochem. Biophys. Res. Commun. 509, 694–699. 10.1016/j.bbrc.2018.12.161 30611569

[B170] Wang K. Liu H. Hu Q. Wang L. Liu J. Zheng Z. (2022a). Epigenetic regulation of aging: implications for interventions of aging and diseases. Signal Transduct. Target Ther. 7, 374. 10.1038/s41392-022-01211-8 36336680 PMC9637765

[B171] Wang W. Chaturbedi A. Wang M. An S. Santhi Velayudhan S. Lee S. S. (2018). SET-9 and SET-26 are H3K4me3 readers and play critical roles in germline development and longevity. Elife 7. 10.7554/eLife.34970 PMC6010342 29714684

[B172] Wang Z. Chivu A. G. Choate L. A. Rice E. J. Miller D. C. Chu T. (2022b). Prediction of histone post-translational modification patterns based on nascent transcription data. Nat. Genet. 54, 295–305. 10.1038/s41588-022-01026-x 35273399 PMC9444190

[B173] Wang Z. Schones D. E. Zhao K. (2009). Characterization of human epigenomes. Curr. Opin. Genet. Dev. 19, 127–134. 10.1016/j.gde.2009.02.001 19299119 PMC2699568

[B174] Wenzel D. Palladino F. Jedrusik-Bode M. (2011). Epigenetics in *C. elegans*: facts and challenges. Genesis 49, 647–661. 10.1002/dvg.20762 21538806

[B175] Whetstine J. R. Nottke A. Lan F. Huarte M. Smolikov S. Chen Z. (2006). Reversal of histone lysine trimethylation by the JMJD2 family of histone demethylases. Cell 125, 467–481. 10.1016/j.cell.2006.03.028 16603238

[B176] Wood J. G. Hillenmeyer S. Lawrence C. Chang C. Hosier S. Lightfoot W. (2010). Chromatin remodeling in the aging genome of Drosophila. Aging Cell 9, 971–978. 10.1111/j.1474-9726.2010.00624.x 20961390 PMC2980570

[B177] Woodhouse R. M. Buchmann G. Hoe M. Harney D. J. Low J. K. K. Larance M. (2018). Chromatin modifiers SET-25 and SET-32 are required for establishment but not long-term maintenance of transgenerational epigenetic inheritance. Cell Rep. 25, 2259–2272.e5. 10.1016/j.celrep.2018.10.085 30463020

[B178] Yi S.-J. Kim K. (2020). New insights into the role of histone changes in aging. Int. J. Mol. Sci. 21. 10.3390/ijms21218241 PMC7662996 33153221

[B179] Yu R. McCauley B. Dang W. (2020). Loss of chromatin structural integrity is a source of stress during aging. Hum. Genet. 139, 371–380. 10.1007/s00439-019-02100-x 31900586 PMC8011432

[B180] Yuan J. Chang S.-Y. Yin S.-G. Liu Z.-Y. Cheng X. Liu X.-J. (2020). Two conserved epigenetic regulators prevent healthy ageing. Nature 579, 118–122. 10.1038/s41586-020-2037-y 32103178

[B181] Zhang H. Kieckhaefer J. E. Cao K. (2013). Mouse models of laminopathies. Aging Cell 12, 2–10. 10.1111/acel.12021 23095062

[B182] Zhang M. Poplawski M. Yen K. Cheng H. Bloss E. Zhu X. (2009). Role of CBP and SATB-1 in aging, dietary restriction, and insulin-like signaling. PLoS Biol. 7, e1000245. 10.1371/journal.pbio.1000245 19924292 PMC2774267

[B183] Zhang T. Cooper S. Brockdorff N. (2015). The interplay of histone modifications - writers that read. EMBO Rep. 16, 1467–1481. 10.15252/embr.201540945 26474904 PMC4641500

[B184] Zhao M. An J. Li H. Zhang J. Li S.-T. Li X.-M. (2017). Segmentation and classification of two-channel *C. elegans* nucleus-labeled fluorescence images. BMC Bioinforma. 18, 412. 10.1186/s12859-017-1817-3 PMC5602880 28915791

[B185] Zhao Y. Wang H. Poole R. J. Gems D. (2019). A fln-2 mutation affects lethal pathology and lifespan in *C. elegans* . Nat. Commun. 10, 5087. 10.1038/s41467-019-13062-z 31704915 PMC6841690

[B186] Zhou J.-J. Chun L. Liu J.-F. (2019a). A comprehensive understanding of dietary effects on *C. elegans* physiology. Curr. Med. Sci. 39, 679–684. 10.1007/s11596-019-2091-6 31612382

[B187] Zhou L. He B. Deng J. Pang S. Tang H. (2019b). Histone acetylation promotes long-lasting defense responses and longevity following early life heat stress. PLoS Genet. 15, e1008122. 10.1371/journal.pgen.1008122 31034475 PMC6508741

[B188] Zhu D. Wu X. Zhou J. Li X. Huang X. Li J. (2020). NuRD mediates mitochondrial stress–induced longevity via chromatin remodeling in response to acetyl-CoA level. Sci. Adv. 6, eabb2529. 10.1126/sciadv.abb2529 32789178 PMC7400466

